# The mitochondrial DNA common deletion as a potential biomarker of cancer-associated fibroblasts from skin basal and squamous cell carcinomas

**DOI:** 10.1038/s41598-023-50213-1

**Published:** 2024-01-04

**Authors:** Gabriele A. Fontana, Michael R. MacArthur, Nadezhda Rotankova, Michela Di Filippo, Hans-Dietmar Beer, Hailey L. Gahlon

**Affiliations:** 1 Department of Health Sciences and Technology, ETH Zurich, 8092 Zurich, Switzerland; 2 Cellvie AG, Technoparkstrasse 1, CH-8005 Zürich, Switzerland; 3 Department of Dermatology, University Hospital Zurich, 8952 Schlieren, Switzerland; 4 Faculty of Medicine, University of Zurich, 8032 Zurich, Switzerland

**Keywords:** Cancer, Biomarkers

## Abstract

Cancer-associated fibroblasts (CAFs) are components of the tumor microenvironment and represent appealing therapeutic targets for translational studies. Conventional protein-based biomarkers for CAFs have been reported to be limited in their specificity, rendering difficult the identification of CAFs from normal fibroblasts (NFs) in clinical samples and dampening the development of CAF-targeted therapies to treat cancer. In this study, we propose the mitochondrial RNA and the mitochondrial DNA (mtDNA) common deletion (CD) as novel indicators of CAF identity. We found that cancer-activation correlated with decreased levels of the mtDNA CD, a condition not due to altered mitochondria count or cellular redox state, but potentially linked to the generalized overexpression of mtDNA maintenance genes in CAFs. Decreased mtDNA CD content in CAFs was associated with moderate to strong overexpression of mtDNA-encoded genes and to slightly improved mitochondrial function. We identified similar patterns of upregulation of mtDNA-encoded genes in independent single-cell RNA seq data obtained from squamous cell carcinoma (SCC) patients. By using the identified nucleic acids-based indicators, identification of CAFs from NFs could be improved, leading to potential therapeutic benefits in advancing translational and clinical studies.

## Introduction

The tumor microenvironment (TME) plays a significant role in the growth and invasion of tumors. Within this complex environment, CAFs are a group of activated fibroblasts that influence tumorigenesis, growth, and metastasis^1^. CAFs are a highly heterogeneous population of cells and are reported to play roles in both tumor suppression^2^ and tumor promotion^3^. In recent years, CAFs have become attractive therapeutic targets due to their immunosuppressive role in the TME^4^. Although there are several clinical trials investigating agents that target CAFs, a current limitation in the field is the absence of specific biomarkers to reliably identify CAFs.

Several conventional biomarkers of CAFs are reported, including α-smooth muscle actin (α-SMA), vimentin, platelet-derived growth factor receptors (PDGFRα/β), fibroblast activation protein (FAP), and S100A4; however, these biomarkers are not specific to CAFs but broadly identify activated fibroblasts. Moreover, these biomarkers cannot discriminate against the high heterogeneity associated with CAFs and their sub-populations^5^. The first identified and most studied CAF biomarker is α-SMA^6,7^, a cytoskeletal protein that forms microfilaments and is associated with the TGF-β pathway. Although studies have shown that α-SMA^+^ CAFs can promote tumor progression and this protein is associated with TME immunosuppression, depleting α-SMA^+^ CAFs in a mouse model did not show promising results as a treatment for pancreatic ductal adenocarcinoma^8^. FAP is another conventional CAF biomarker, and FAP^+^ CAFs are reported to have an immunosuppressive role in the TME by means of cytokine and chemokine secretion^9–11^. While FAP is one of the most promising CAF targets as a druggable target for anticancer therapy, still more studies are needed to elucidate its role in tumor progression and suppression.

To advance the therapeutic potential of targeting CAFs, new biomarkers are needed to identify and discriminate them within heterogenous populations. Reports have shown that CAF number and CAF function are associated with patient outcome^12,13^. The ability to reprogram or eliminate CAFs, or block signals directly from CAFs to the surrounding TME are viable strategies that could provide clinical benefit. Still, to advance the therapeutic potential for CAF-targeting agents, new biomarkers to specifically identify CAFs are needed. In this work, we report, for the first time, the mitochondrial common deletion and mitochondrial-encoded genes as potential biomarkers to identify CAFs in both squamous cell carcinoma (SCC) and basal cell carcinoma (BCC) tissue. These data provide the possibility for new biomarkers that could significantly advance the field by providing new tools to identify and study CAF-associated tumor biology and function.

## Results

### CAFs show reduced mtDNA common deletion heteroplasmic levels compared to NFs

**Figure 1:**
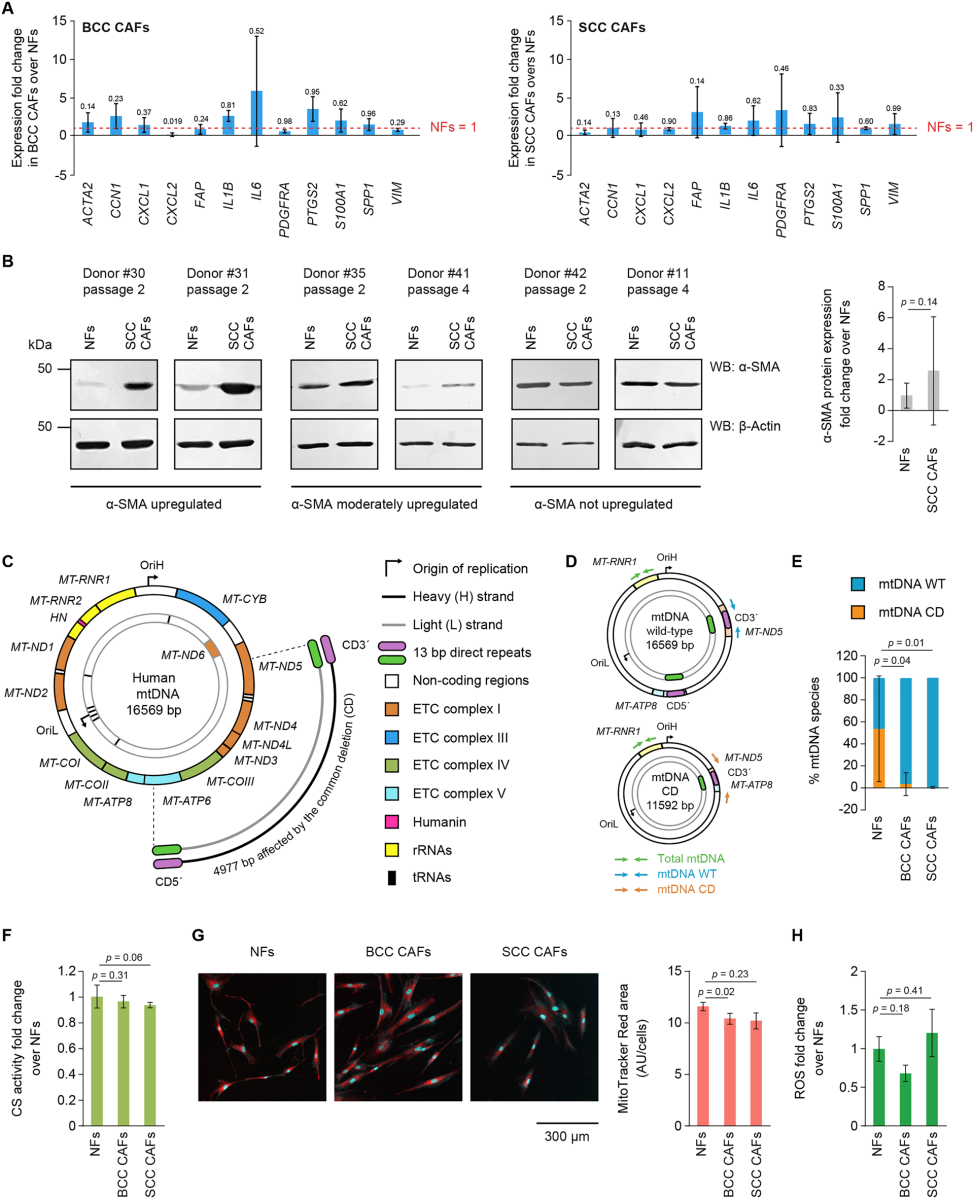
BCC and SCC CAFs harbor decreased levels of the mtDNA CD as compared to NFs. (**A**) Evaluating expression of conventional CAF biomarkers in BCC CAFs (left) and SCC CAFs (right) by qPCR. The expression levels of the indicated genes were expressed as fold change as compared to NFs. n = 3 biological replicates, statistical analyses: unpaired Wilcoxon *t* test for BCC CAFs, paired Mann–Whitney *t* test for SCC CAFs. (**B**)WBs for the expression of α-SMA in NFs and SCC CAFs from the same donors. Left: representative WBs from donor-matched NFs and SCC CAFs at low passages (P2 and P4). Right: quantitation of α-SMA levels from n = 15 NFs and SCC CAFs pairs. The expression of α-SMA in SCC CAFs is expressed as fold change as compared to NFs, variability is depicted as SD, statistical analysis: paired *t* test. (**C**) Human mtDNA, containing genes encoding proteins of the electron transport chain (ETC), Humanin, rRNAs and tRNAs. The ~ 5 kb region affected by the common deletion (CD) is flanked by two 13 bp direct repeats. (**D**) Schematic representations of WT and CD mtDNA molecules showing localization of the primers for the analysis of total., WT and CD mtDNA copies within the mitochondrial genome. (**E**) qPCR for the relative quantification of mtDNA WT and CD in NFs, BCC CAFs and SCC CAFs. n = 6 biological replicates. (**F**) Citrate synthase (CS) activity in NFs, BCC CAFs and SCC CAFs. The CS activity in BCC and SCC CAFs is expressed as fold change as compared to NFs. n = 3 biological replicates. (**G**) Left*:* representative microscopic images of NFs, BCC and SCC CAFs stained with Mitotracker Red for membrane polarization and DAPI for nuclei identification. Right: quantification of total mitochondrial area normalized for cell count. *AU* arbitrary units. n = 3 biological replicates. (**H**) Measurement of ROS in NFs, BCC CAFs and SCC CAFs by DCF assay. n = 3 biological replicates. For panels (**E**–**H**) statistical analysis was Kruskal–Wallis test with Dunn’s post hoc test comparing to NFs.

Conventional biomarkers for CAFs display inconsistent expression differences as compared to normal fibroblasts (NFs) and do not reliably discriminate healthy from cancer-activated cells. We corroborated these observations by analysing the transcriptional levels of twelve biomarkers commonly associated with cancer activation of fibroblasts in primary NFs and CAFs derived from BCC and SCC patients (Fig. 1A). qPCRs confirmed that the selected biomarkers do not show significant alterations in their transcription in BCC and SCC CAFs as compared to patient-matched NFs. While genes encoding inflammatory markers such as *IL1B* and *IL6* show an upregulation trend in CAFs as compared to NFs (*IL1B*: ~ 2.5 and ~ 1.2 average fold change in BCC and SCC CAFs, respectively; *IL6*: ~ 5.8 and ~ 1.9 average fold change in BCC and SCC CAFs, respectively), the high variability among biological replicates of NFs and CAFs reduced power to detect statistical significance. We further validated the transcriptional data by analysing protein levels of a widely used CAF biomarkers, the α-SMA protein encoded by the *ACTA2* gene, in fifteen patient-matched NFs and SCC CAFs pairs (Fig. 1B). The protein levels of the α-SMA protein were highly variable among NFs and SCC CAFs pairs; in fact, we identified donors where α-SMA was strongly or moderately upregulated in CAFs and donors where protein levels were comparable between NFs and CAFs. While the quantitation of expression levels showed a ~ 2.6 average fold upregulation of α-SMA in SCC CAFs as compared to NFs, the subsequent statistical analysis revealed high variation and no significant difference between the α-SMA protein amounts in CAFs (Fig. 1B).

Towards the aim of finding novel, robust and specific biomarkers for CAFs, we evaluated a specific 4977 bp deletion named the common deletion (CD, Fig. 1C) for its widespread association with diverse conditions, including cancer^14,15^. Heteroplasmic levels of the CD among mtDNA WT molecules increase during aging in multiple tissues^16,17^ and altered mtDNA CD heteroplasmic contents have been linked to severe mitochondrial pathologies^18,19^ and have been observed in tumor cells of diverse origin^20,21^. The ~ 5 kb region affected by the CD is flanked by two 13 bp direct repeats, which have been proposed to play mechanistic roles in the generation of this mutational event potentially via their mispairing during replication-coupled mtDNA repair^22^. We developed a qPCR-based strategy to monitor the relative heteroplasmic levels of the mtDNA WT and CD species in mtDNA purified from primary NFs and CAFs (Fig. 1D). By detecting amplicons of deleted and undeleted mtDNA species and relating them to the total amount of mtDNA in the sample, we expressed relative percentages of mtDNA WT and CD in SCC and BCC CAFs (Fig. 1E). We found that NFs contain an average of ~ 53% of mtDNA CD molecules, albeit with significant variation due to inherent variability of donor-derived primary cells. Strikingly, we found that in patient-matched BCC and SCC CAFs the levels of the CD are significantly lower, reaching averages of ~ 4.6% in BCC CAFs and ~ 0.5% in SCC CAFs, with less variation than NFs.

The dramatic difference in the mtDNA WT to CD ratio observed between NFs and CAFs prompted us to investigate whether this was due to altered mitochondrial mass. We therefore measured citrate synthase (CS) activity, an enzyme localized within the mitochondrial matrix, whose activity is proportional to the amount of intact cellular mitochondria. We did not detect changes in CS activity between NFs, BCC and SCC CAFs, suggesting that cancer-activation does not induce alterations in the mitochondrial content of fibroblasts (Fig. 1F). To further corroborate this observation, we imaged NFs and CAFs stained with MitoTracker Red, a fluorescent dye that detects intact and functional mitochondria, and DAPI, providing nuclear staining for parallel cell count (Fig. 1G). Microscopic analysis did not reveal significant differences in the amounts of mitochondria per cell, as measured by the ratio between the cumulative area of the MitoTracker Red staining and the total amount of cells in the monitored fields. Variations in the levels of the mtDNA CD have been correlated with oxidative stress and the mutagenic effects of reactive oxygen species (ROS) on the mtDNA^23^; thus, we also monitored the basal levels of total cellular ROS in NFs and CAFs using the established 2′,7′-Dichlorofluorescin diacetate (DCF) fluorescent reporter assay. We did not detect alterations in the basal ROS content in NFs and CAFs (Fig. 1H).

### Several mtDNA maintenance genes are upregulated in primary BCC and SCC CAFs

**Figure 2:**
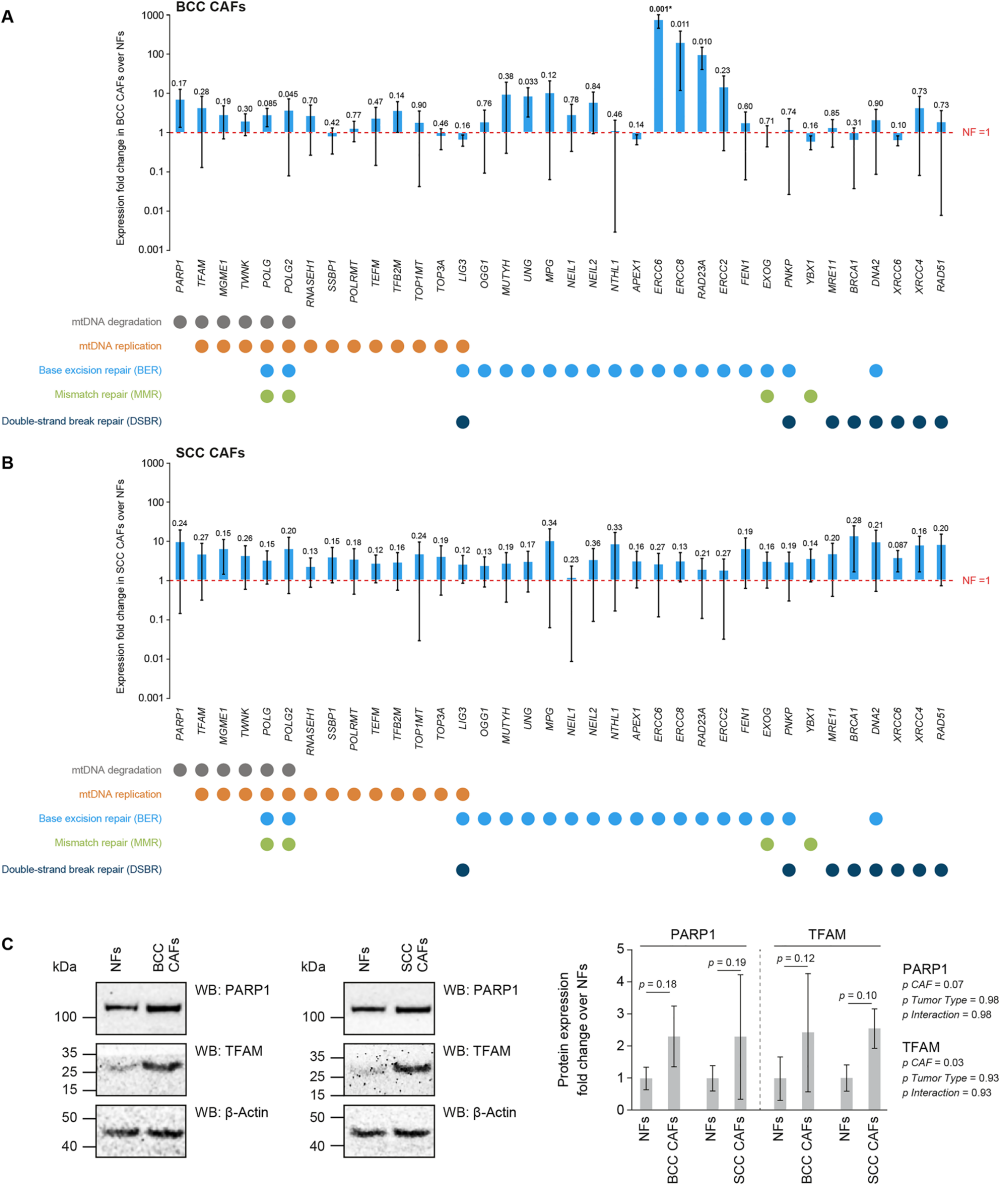
Expression of key genes involved in mtDNA maintenance is upregulated in BCC and SCC CAFs. qPCR analysis for the expression of nuclear DNA-encoded genes involved in mtDNA maintenance processes, i.e. mtDNA degradation, replication and repair (see legends at the bottom of panels). Experiments were performed in BCC CAFs (the asterisk indicates a comparison surviving Benjamini–Hochberg False Discovery Rate correction) (**A**) and SCC CAFs (**B**), and changes in expression levels were expressed as fold changes on a log scale over matching NFs. n = 3 biological replicates were used. Statistical analysis was performed with an unpaired Wilcoxon *t* test for BCC CAFs and with a paired Mann–Whitney *t* test for SCC CAFs. (**C**) WBs were performed to monitor the expression levels of PARP1 and TFAM protein in NFs, BCC and SCC CAFs. Left: representative WBs panels. Right: quantitation of WBs for PARP1 and TFAM. Expression levels in BCC and SCC CAFs are expressed as fold change as compared to NFs. n = 3 biological replicates were used. Statistical analysis for BCC CAFs was conducted with a Mann Whitney unpaired *t* test and for SCC CAFs with a paired Wilcoxon *t* test.

We measured by qPCR the transcriptional levels of a panel of candidate genes encoding proteins involved in mtDNA replication, repair and degradation; all mechanisms whose dysfunctions have been linked to mtDNA CD formation and maintenance^14^. We found that the majority of the interrogated genes show an upregulation trend in both BCC CAFs (Fig. 2A) and SCC CAFs (Fig. 2B) as compared to NFs. In particular, the expression of *TFAM*, encoding an mtDNA-binding protein organizing nucleoids and playing crucial roles in the maintenance of the mitochondrial genome, is upregulated in both CAF population (average fold change of ~ 4.2 in BCC CAFs and ~ 16.6 in SCC CAFs). Similarly, genes encoding regulators of mtDNA replication, including *MGME1* (average fold change of ~ 2.7 in BCC CAFs and ~ 10.6 in SCC CAFs)*, TWNK* (average fold change of ~ 1.9 in BCC CAFs and ~ 9.5 in SCC CAFs), *POLG* (average fold change of ~ 2.7 in BCC CAFs and ~ 11 in SCC CAFs), *POLG2* (average fold change of ~ 3.6 in BCC CAFs and ~ 10.8 in SCC CAFs), *TEFM* (average fold change of ~ 2.2 in BCC CAFs and ~ 9.9 in SCC CAFs) *TFB2M* (average fold change of ~ 3.5 in BCC CAFs and ~ 9.7 in SCC CAFs) and *TOP1MT* (average fold change of ~ 1.7 in BCC CAFs and ~ 9.2 in SCC CAFs) are consistently upregulated in both CAF populations. The expression of genes encoding enzymes participating in the base excision repair (BER) pathway (see Fig. 2A,B for associated BER genes, indicated in blue circles), the only mtDNA repair mechanism proven to occur in mitochondria in vivo, also displayed an upregulation in both CAF populations. The expression of proteins involved in the mismatch repair and double-strand break repair pathways, two repair mechanisms with little in vivo evidence to play a role in mitochondria, display diverse patterns of up- and down-regulation between BCC and SCC CAFs.

To test whether transcriptional upregulation would lead to a concomitant increase in protein levels, we assessed the levels of PARP1 and TFAM, two factors that play key roles in mtDNA maintenance^24,25^ in NFs and CAFs (Fig. 2C). In good agreement with the qPCR data, we found upregulation of the protein levels of PARP1 (average fold change of ~ 2.3 in BCC and SCC CAFs) and TFAM (average fold change of ~ 2.4 in BCC CAFs and ~ 2.6 in SCC CAFs) in both CAF populations.

### mtDNA-encoded gene expression is altered in primary BCC and SCC CAFs, leading to moderately improved mitochondrial metabolism

**Figure 3:**
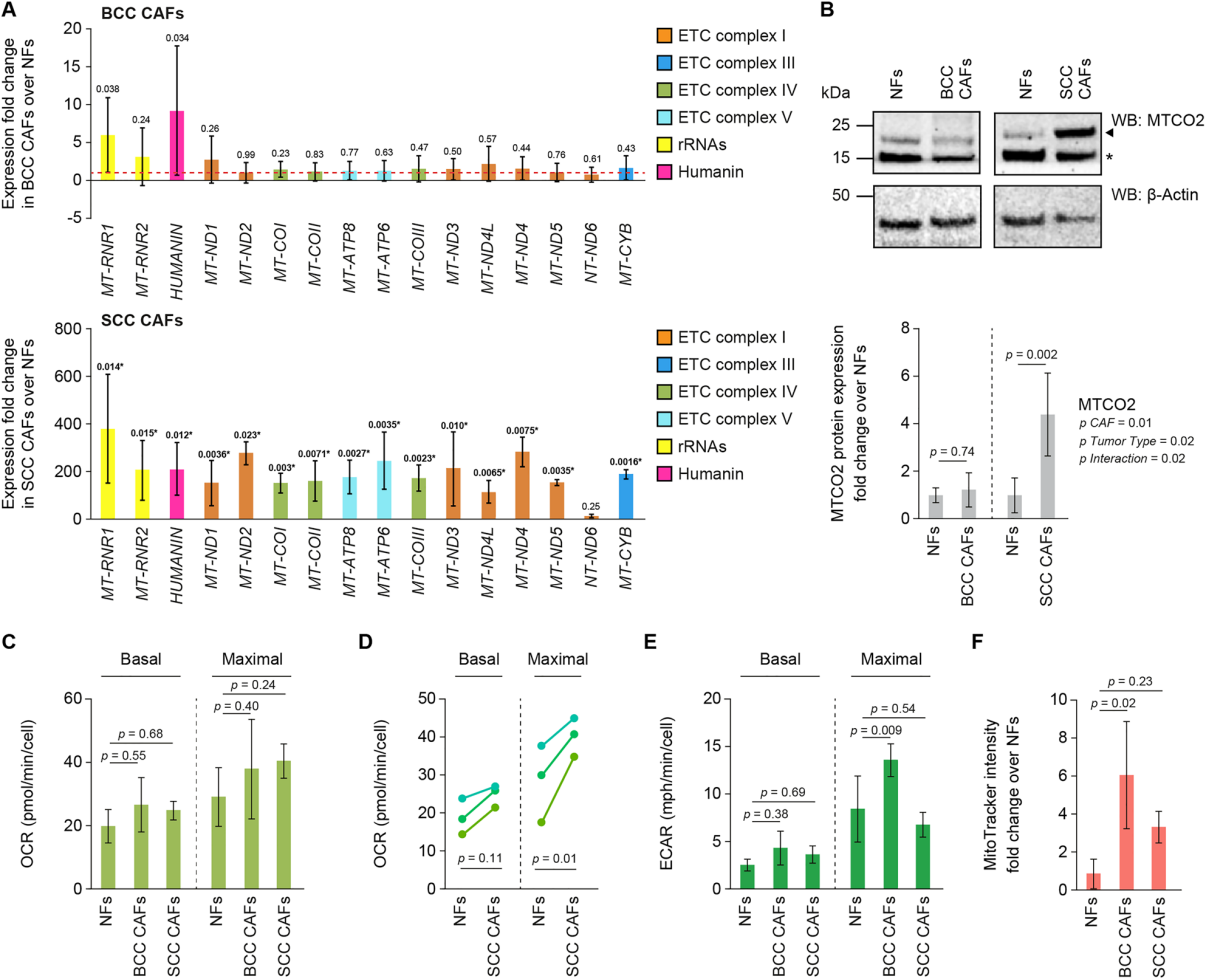
Expression of mtDNA protein-coding genes is upregulated in BCC and SCC CAFs. (**A**) qPCR analysis for the expression of mtDNA-encoded genes in BCC CAFs (upper panel) and SCC CAFs (bottom panel), expressed as fold change over isogenic NFs (red dotted line). Color codes indicate the ETC complexes to which the encoded proteins belong to, rRNAs and the Humanin micropeptide. n = 3 biological replicates were used for each graph. Statistical analysis was performed with an unpaired Wilcoxon *t* test for BCC CAFs and with a paired Mann–Whitney *t* test for SCC CAFs (the asterisk indicates a comparison surviving Benjamini–Hochberg False Discovery Rate correction). (**B**) WB analysis for the protein levels of MTCO2 in BCC and SCC CAFs. Upper panel: representative WBs. Arrowhead indicates MTCO2, asterisk designates a cross-reactive unspecific band. Bottom panel: quantitation of MTCO2 expression in n = 3 biological replicates, expressed as fold change over NFs. Statistical analysis was performed with a Wilcoxon *t* test. (**C**) Oxygen consumption rates (OCR) at basal and stimulated (maximal) ETC activity in NFs, BCC and SCC CAFs. n = 3 biological replicates were used and analyzed by Kruskal–Wallis test with Dunn’s post hoc test. (**D**) OCR in basal and maximal conditions in three patient-matched NFs and SCC CAFs pairs analyzed by repeated measures two-way ANOVA with Sidak post-hoc test. (**E**) Extracellular acidification rates (ECAR) at basal and maximal ETC activity in NFs, BCC and SCC CAFs. Results relative to n = 3 biological replicates and analyzed by repeated measures 2-way ANOVA with Sidak post-hoc test. (**F**) Quantification of the intensity of Mitotracker Red staining normalized for cell count. Data are expressed as fold change over NFs and derive from n = 3 biological replicates. Analyzed by Kruskal–Wallis test with Dunn’s post hoc test.

Having detected a decrease in the mtDNA CD content and a concomitant increase in the mtDNA WT proportion in BCC and SCC CAFs as compared to NFs, we sought to determine the expression levels of mtDNA-encoded genes and the functionality of the electron transport chain (ETC) machinery. We initially tested by qPCR the transcriptional levels of mtDNA-encoded genes and found different trends between two CAF types (Fig. 3A). In BCC CAFs, most mtDNA-encoded genes did not show altered expression, with the exceptions of the *MT-RNR1* and *MT-RNR2* genes, encoding the mitochondrial-specific 12s and 16s ribosomal RNA subunits, and the Humanin open reading frame, encoding a micropeptide involved in stress responses and inflammation, which show an average fold change of ~ 6.0, 3.1 and ~ 9.3 as compared to NFs, respectively. In SCC CAFs, all the mtDNA-encoded genes showed a marked upregulation as compared to NFs, with average fold changes ranging from ~ 15 of *MT-ND6* and ~ 380 of *MT-RNR1*.

It has been shown that the levels of polycistronic RNA transcribed from mtDNA molecules do not strictly correlate with increased translation of the corresponding encoded proteins^26^. To test whether the transcriptional levels of the mtDNA-encoded genes assessed by qPCR translates into similar patterns of protein expression, we monitored by WB the levels of MTCO2, encoded by the *MT-CO2* mtDNA gene, in NFs and CAFs (Fig. 3B). In agreement with the transcriptional data, we found that MTCO2 expression is unaltered in BCC CAFs and upregulated in SCC CAFs as compared to NFs.

Having detected differences in the expression patterns of mtDNA-encoded genes in the two populations of CAFs as compared to NFs, we then sought to perform functional assays to determine the activity of the ETC and the overall rates of mitochondrial metabolism in NFs and CAFs. Using the established Seahorse assay we simultaneously measured the oxygen consumption rates (OCR) and extracellular acidification rate (ECAR), monitoring mitochondrial respiration and glycolysis in live cells, respectively. The OCR was determined in both basal conditions, i.e. before addition of chemical modulators of the activity of the ETC, and maximal conditions, i.e. after addition of the protonophore FCCP to collapse the inner mitochondrial membrane gradient (Fig. 3C). We found that both BCC and SCC CAFs display slightly increased OCR as compared to NFs in basal state and more marked increased OCR in maximal conditions, albeit with high variability among biological replicates. Comparing patient-matched NFs and SCC CAFs pairs showed a significant effect of cancer status (Fig. 3D). We also monitored the ECAR, which arises from acidification of the culture medium due to lactate diffusion in the culture medium as a proxy for glycolytic activity. The ECAR was monitored in both basal conditions, i.e. before addition of chemical modulators of activity of the ETC, and maximal conditions, i.e. after addition of FCCP (Fig. 3E). ECAR quantifications revealed a significant effect of cell type (2-way ANOVA *P* = 0.006) but no significant differences in basal rate upon post-hoc testing. Upon FCCP treatment BCCs had a significantly higher rate compared to NF and SCC (Fig. 3E). As an orthogonal measure of mitochondrial metabolism, we monitored the mitochondrial membrane potential by quantifying the intensity of the internalized MitoTacker Red staining normalized on a per cell basis (Fig. 3F). While both CAF populations tended have higher membrane potential, only BCC was significantly higher.

### Single-cell data from SCC CAFs reveals upregulation of mtDNA-encoded genes

**Figure 4:**
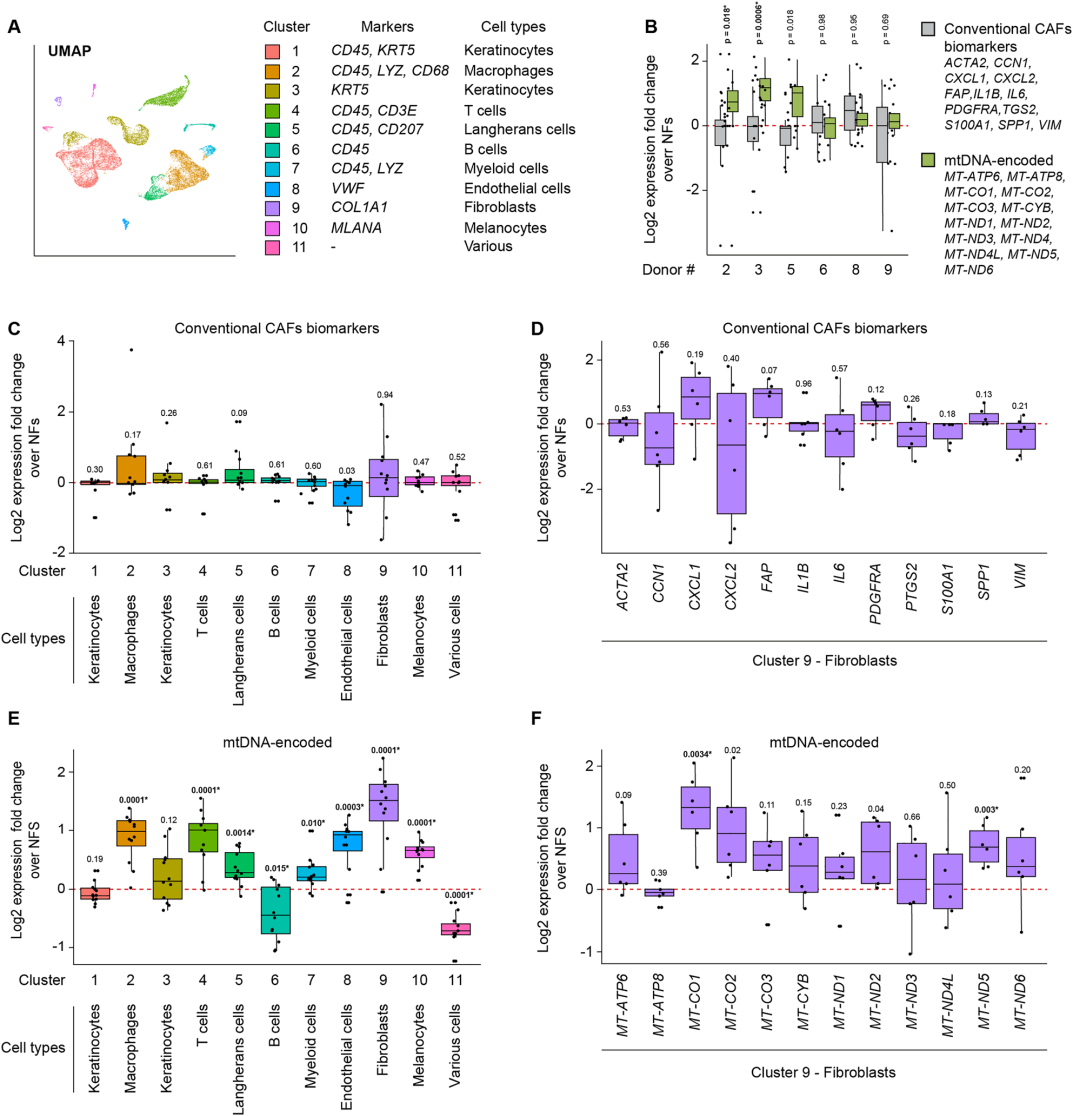
Single cell sequencing data corroborates the upregulation of mtDNA-encoded genes in SCC CAFs. (**A**) UMAP analysis of single-cell sequencing data collected by Ji et al.^27^ from primary SCC CAFs and matched NFs from twelve donors. Eleven clusters were identified based on the expression of the gene markers listed in the legend. The fibroblasts cluster, accounting for ~ 5% of the sequenced cells, was analyzed further. (**B**) Analysis of the expression of conventional CAF biomarkers (grey bars, genes indicated in the legend) and mtDNA-encoded genes (green bars, genes indicated in the legend) in NFs and SCC CAFs from six patients with sufficient fibroblasts sequenced. NFs were set to 1 and relative expression fold changes in Log2 scale were calculated for each NFs and CAF pair (the asterisk indicates a comparison surviving Benjamini–Hochberg False Discovery Rate correction). (**C**) Relative expression of conventional CAF biomarkers in the eleven clusters identified by the UMAP analysis. (**D**) Relative expression of twelve conventional CAF biomarkers analyzed in the UMAP cluster 9, i.e. the fibroblasts cluster. (**E**) Relative expression of mtDNA-encoded genes in the eleven clusters identified by the UMAP analysis. (**F**) Relative expression of mtDNA encoded genes analyzed in the UMAP cluster 9, i.e. the fibroblasts cluster.

We analysed single-cell RNA sequencing data from a previous study^27^ that compared patient-matched NFs and SCC CAFs. To separate the fibroblasts pool among the mixed cell population contained in the datasets we performed clustering and UMAP projection, clustering sequenced cells based on expression of single and/or combinations of gene markers (Fig. 4A). This analysis retrieved eleven cellular clusters of similar composition to the original publication. We observed that in samples from healthy skin regions and patient-matched cancer-proximal tissues the fibroblasts pool was limited, representing ~ 5% or less of total cells. Among the twelve SCC patients from which cells where sampled, we identified six patients where the population of sequenced fibroblasts was sufficiently represented, allowing subsequent analyses. We profiled the cumulative expression of twelve conventional CAF biomarkers and mtDNA-encoded genes in the six patients (Fig. 4B). This analysis revealed that the expression of the selected conventional CAF biomarkers was highly variable and did not consistently differ between CAFs and matched NFs. Conversely, we detected a more consistent upregulation trend in the cumulative expression of mtDNA-encoded genes in CAFs of the six patients.

To evaluate potential correlations between the expression of the genes of interest and cancer-activation, we monitored the expression of conventional CAF biomarkers and mtDNA-encoded genes in a cumulative fashion within the cell clusters identified by the UMAP analysis or as single entities within the fibroblasts cluster and within samples derived from NFs and SCC CAFs. The cumulative analysis of conventional CAF biomarkers in the eleven cell clusters derived from the UMAP analysis revealed that these genes do not display marked upregulation trends in any clusters (Fig. 4C). When individual genes were in the fibroblast cluster, *CXCL1*, *FAP* and *PDGFRA* displayed moderately higher levels in SCC CAFs as compared to NFs with high variability (Fig. 4D). We repeated these analyses for the mtDNA-encoded genes. By monitoring their cumulative expression in the eleven UMAP-identified clusters, we found that in the skin, immune and endothelial cells clusters the mtDNA-encoded genes were upregulated; interestingly, the highest degree of upregulation was observed in the fibroblasts cluster (Fig. 4E). The analysis of mtDNA-encoded genes within the fibroblast cluster revealed that multiple genes, especially *MT-CO1*, *MT-CO2* and *MT-ND5*, were upregulated in SCC CAFs of all patients (Fig. 4F).

## Discussion

The CD as a biomarker for tumorigenesis has been studied^15^; in some cancers, the CD is highly abundant, while in others it is not detected. Accumulation of the CD has been consistently linked to the emergence of multisystemic mitochondrial pathologies, such as the Kearns–Sayre syndrome (KSS)^28,29^ and progressive external ophthalmoplegia (PEO)^29^. KSS and PEO encephalomyopathies manifest dysfunctions in the nervous, muscular and endocrine system. On a molecular level, cells derived from KSS and/or PEO patients show elevated levels of CD heteroplasmy, accompanied by dysfunctional mtDNA gene expression, impaired oxidative phosphorylation and a decrease in cellular energy production^30,31^. Potential correlations between variable CD levels in CAFs were not so far reported. We find that in SCC and BCC CAFs, the CD levels are significantly lower in comparison to NFs (Fig. 1E), suggesting that the CD is a potential molecular biomarker to discriminate CAFs from NFs. To our knowledge this observation has not been previously reported. Previous studies link increased CD levels to aging^16,17^; therefore, the observed elevated levels of the CD in NFs as compared to SCC and BCC CAFs could be explained by the fact that our NFs and corresponding CAFs derive from aged patients. While the molecular mechanism of CD formation and maintenance is not well understood, it has been reported that ROS is associated with its formation^23^. However, we did not detect alterations in the ROS basal content in NFs and CAFs (Fig. 1H), suggesting that oxidative stress may not play a key role in determining the diverse heteroplasmic contents of the CD between NFs and CAFs.

Seeking potential explanations for the decreased levels of the mtDNA CD in BCC and SCC CAFs and compared to NFs, we hypothesized that cancer activation could reprogram the expression patterns of genes encoding mtDNA maintenance proteins. Following this rationale, CAFs would prevent the formation of the CD or degrade mtDNA molecules which acquire the CD. To investigate this hypothesis, we measured by qPCR the transcriptional levels of a panel of candidate genes encoding proteins involved in mtDNA replication, repair and degradation (Fig. 2A,B), all mechanisms whose dysfunctions have been linked to mtDNA CD formation^14,32^. We found that the majority of the interrogated genes showed an upregulation trend in both SCC and BCC CAFs compared to NFs. These data suggest that the expression of genes encoding proteins involved in mtDNA maintenance (i.e. replication, degradation and BER), all pathways whose dysfunctions have been linked to the emergence of mtDNA deletions and the mtDNA CD, is upregulated in CAFs. This observation could be explained by hyperactive mtDNA maintenance pathways in CAFs, thereby resulting in improved clearance and/or reduced formation of the CD in CAFs as compared to NFs.

Mitochondrial-encoded genes have not yet been reported as biomarkers for CAFs. We observed a general increase in transcriptional levels of mtDNA-encoded genes for BCC and SCC CAFs (Fig. 3A,B, respectively). Surprising to us, the SCC CAFs had very significant overexpression as compared to NFs (e.g. ~ 380-fold increase of *MT-RNR1*), much more so than for the BCC CAFs. This observation could be explained by the fact that SCC often is associated with a stronger cancer phenotype in comparison to BCC. For example, BCC cells tend to grow less invasively and are attributed to less severe clinical consequences than SCC^33,34^. In our functional assays for mitochondrial metabolism, we observed a moderate increase in the OCR and ECAR in both SCC and BCC CAFs compared to NFs (Fig. 3C–E). Tumor growth is associated with altered mitochondrial metabolism and the modulation of oxygen consumption is reported as a rate limiting substrate for tumorigenesis^35^. In skin cancer, as well as most cancer types, hypoxic conditions in precancerous tissue promote cancer formation^36^. This might explain the observation for increased OCR in our SCC and BCCs, such that as cancer develops and tumor mass increases, oxygen consumption will increase due to the rise in actively proliferating cells^37^. Taken together, these findings suggest that the elevated heteroplasmic content of wild-type mtDNA molecules in CAFs as compared to NFs lead to increased transcription of mtDNA-encoded genes, with significant differences of expression rates among BCC and SCC CAFs. This translates in moderately increased mitochondrial activity, suggesting that the suppression of the deleted mtDNA population observed in CAFs may modestly affect mitochondrial metabolism.

Finally, we were pleased to observe that in an independent study that previously reported single-cell RNA sequencing data in SCC patients^27^, a corroboration to our qPCR data showing the upregulation of mitochondrial-encoded genes in SCC CAFs (Fig. 3A). The fact that we could observe an overall upregulation for most mitochondrial genes in the fibroblast cluster, suggests that this phenotype may be ascribed to SCC CAFs down to the single cell level.

In summary, our findings show that primary BCC- and SCC-derived CAFs display significantly reduced contents of the mtDNA CD as compared to isogenic NFs. In both SCC and BCC CAFs, we consistently observed a pattern of upregulation in genes involved in mtDNA maintenance; thus, molecular mechanisms selectively removing and/or preventing the formation the mtDNA CD could be hyperactive in CAFs. In addition, SCC and BCC CAFs with increased WT mtDNA levels displayed enhanced expression of mtDNA-encoded genes and moderately improved mitochondrial functionality. We validated the increased expression of the mtDNA-encoded genes by evaluating independent single-cell RNA sequencing data from SCC CAFs, which corroborated our qPCR data. In conclusion, we propose that the mtDNA CD heteroplasmic content and the expression of mtDNA-encoded genes represent the potential for robust and specific biomarkers to discriminate CAFs from NFs. These suspected biomarkers may hold promise to advance clinical and translational studies aimed to understand CAF function and biology.

## Materials and methods

### Patients and clinical samples

Fibroblasts from basal cell carcinoma (BCC) and normal fibroblasts (NFs) from healthy skin were obtained from the Biobank of the dermatology department of the University Hospital Zurich managed by SINTEGRITY.CH research program. Fibroblasts from squamous cell carcinoma (SCC) and NFs from healthy skin were obtained from Beer laboratory at the University Hospital Zurich. SCCs and surplus human biopsies used to isolate cells were collected after obtaining patient informed written consent and upon approval from local ethical committees and were conducted according to the principles of the Declaration of Helsinki. The information relative to patients are reported in Table S1.

### Primary fibroblasts cultures

Normal fibroblasts (NFs) and CAFs were isolated from the skin of healthy and BCC or SCC patients using the procedure reported in^38–41^. Isolated cells were then cultured in Dulbecco’s modified Eagle medium (DMEM), high glucose, pyruvate (ThermoFisher Scientific, USA) with 10% fetal bovine serum (FBS, ThermoFisher Scientific, USA). For BCC CAFs and associated NFs the medium was supplemented with 100 μg/ml Normocin (Invivogen, USA). For SCC and associated NFs the medium was supplemented with 100 units/ml of Penicillin and 100 µg/ml of Streptomycin (ThermoFisher Scientific, USA). Cells were routinely passaged every 2–3 days in 10 cm Petri dishes and/or T75 flasks and detached with a solution of Trypsin–EDTA 0.25% and phenol red (ThermoFisher Scientific, USA). Experiments were performed at passages comprised between 2 and 6.

### RNA and mtDNA extraction

Cells were grown to ~ 70–80% confluency, harvested by scraping and pelleted by centrifuging. RNA was isolated from cells pellets from NFs, BCC and SCC CAFs using the RNeasy Mini Kit (Qiagen, Germany) following manufacturer’s instructions. The RNA preparation included an on-column RNase-Free DNase (Qiagen, Germany) treatment prior to elution. mtDNA was isolated from cells pellets using the protocol described in^42^ with minor changes. Briefly, cell pellets were resuspended in 250 µl Buffer P1 from the QIAprep Spin Miniprep Kit (Qiagen, Germany) supplemented with RNAse A. The resuspended cells were sonicated using a Vibra-Cell Ultrasonic Liquid Processor (Sonics and Material, model VCX750, USA) with three pulses of 30 s each, at maximum amplitude, with intervals of 2 min in ice. The mtDNA was isolated following the QIAprep Spin Miniprep Kit instructions, and further purified using the Agencourt AMPure XP (Beckman Coulter, USA) paramagnetic beads. Concentrations of purified RNA and mtDNA was assessed using a NanoDrop (ThermoFisher Scientific, USA) spectrophotometer.

### Quantitative real-time PCR (qPCR) for the analysis of gene expression levels

1–2 µg of purified RNA were retrotranscribed to cDNA using the High-Capacity cDNA Reverse Transcription Kit (ThermoFisher Scientific, USA) following manufacturer’s instructions. Each qPCR sample consisted in 4–6 ng of cDNA, 5 × HOT FIREPol EvaGreen qPCR Mix (Solis Biodyne, Estonia), 0.4 μM forward primer, 0.4 μM reverse primer and nuclease-free water to a total of 12 µl. The primers used in this study are listed in Table S2 and were obtained from Eurogentec, Belgium. qPCRs were conducted using a Rotor-Gene 6000 (Corbett Research, Australia) device, using the following protocol for each cycle: 95 °C for 30 s, 60 °C for 1 min, 40–50 cycles, with fluorescence acquisition at the end of the last amplification step. The expression levels for nuclear genome-encoded target genes were assessed in each biological condition using the delta-delta Ct method using the expression of *GAPDH* or *ACTB* housekeeping genes as normalizers. The expression levels for mtDNA-encoded target genes were assessed in each biological condition using the delta-delta Ct method using the expression of an untranslated region of the polycistronic mtDNA-encoded RNAs as normalizer. Each combination of cDNA and primers was assayed in three technical replicates. The number of biological replicates relative to each experiment are indicated in the Figure legends.

### Quantitative real-time PCR (qPCR) for the analysis of mtDNA species

5–10 ng of purified and RNA-free mtDNA was assayed by qPCR using the GoTaq 2 × qPCR Master Mix (Promega, USA), 0.4 μM forward primer, 0.4 μM reverse primer and nuclease-free water to a total of 12 µl per sample. The primers used in this study are listed in Table S2 and were obtained from Eurogentec, Belgium. qPCRs were conducted using the Rotor-Gene 6000 (Corbett Research, Australia) device, following a standard qPCR protocol. Normalization was performed with the delta-delta Ct method using the qPCR data for the amplification of the total mtDNA content in each sample as normalizer. The corresponding values obtained from the qPCR for the mtDNA CD and undeleted species were expressed as relative fractions over the corresponding total mtDNA. The levels of genomic DNA contamination in the mtDNA preparation were routinely assessed by performing parallel qPCRs on a genomic region of the *ACTB* gene, resulting in low or absent signal. Each combination of mtDNA and primers was assayed in three technical replicates. The number of biological replicates relative to each experiment are indicated in the figure legends.

### Western blotting

NFs, BCC and SCC CAFs were grown to confluency of ~ 80%, and procedures for western blotting followed two protocols. For the western blotting depicted in Fig. 1B, cells were collected by scraping in SDS loading buffer composed as follows: 50 mM Tris–HCl pH 6.8, 2% SDS, 10% Glycerol, 0.025% Bromophenol Blue, supplemented with freshly added 100 mM DTT (all reagents from Sigma-Aldrich, USA). Cells were resuspended, boiled and sonicated. Proteins were separated on SDS-PAGE gels with Mini-PROTEAN Tetra vertical electrophoresis cell (Bio-Rad Laboratories Inc., USA) alongside a Prestained Protein MW Marker (ThermoScientific, USA) and transferred to an Amersham Protran 0.2 µm nitrocellulose membrane (GE Healthcare, USA). The membranes were blocked for 1 h at room temperature with 3% non-fat dried milk in PBS supplemented with 0.5% Tween20 (Sigma-Aldrich, USA) and incubated overnight at 4 °C with primary antibodies diluted in PBS-0.5% Tween20 supplemented with 3% non-fat dried milk. After extensive washes in PBS-0.5% Tween20, secondary antibodies were incubated for 1 h at room temperature. Proteins were detected using BCIP/NBT Color Development Substrate (Promega, USA) and membranes were scanned using a LiDE210 scanner (Canon, Japan). Band intensity was quantified using ImageJ software (NIH, USA) and normalized to β-Actin levels. A second western blot was performed with normalized protein amounts and used for subsequent quantifications and assembly of the panels. For the western blots depicted in Figs. 2C and 3B, cells were harvested by scraping and cell pellets were washed with cold PBS. Whole cell lysates were obtained by lysis with RIPA Lysis and Extraction Buffer (ThermoFisher, USA) supplemented with Complete Mini EDTA-free Protease Inhibitor Cocktail (Roche, Switzerland). Protein concentration was assessed with the Pierce BCA protein assay kit (ThermoFisher, USA) and 20 µg of WCE were loaded on a NuPAGE 4–12%, Bis–Tris, 1.0 mm, Mini Protein Gel, 10-well (Invitrogen, USA), alongside the PageRuler Prestained Protein Ladder (ThermoFisher, USA). Electrophoretic run was performed using the MES SDS Running Buffer (ThermoFisher, USA). Proteins were blotted on the Trans-Blot Turbo mini-size PVDF membrane (Bio-Rad Laboratories Inc., USA). The membrane was blocked for 1 h with 5% non-fat dry milk diluted in Tris-buffered Saline Buffer supplemented with 0.1% Tween20 (TBS-T). After extensive washing in TBS-T, membranes were incubated with primary antibodies diluted in TBST-T with 5% dry milk and incubated overnight at 4 °C. After extensive washing with TBS-T, incubation with secondary antibodies diluted in TBST-T with 5% dry milk for 1 h at room temperature. After washing, the membrane was incubated with the ECL Western Blotting Substrate (Promega, USA) and signals were acquired with a ChemiDoc MP imaging system (Bio-Rad Laboratories Inc., USA). Quantification of bands was performed using the Fiji software^43^. The number of quantified biological replicates are indicated in the figure legends. The primary and secondary antibodies used in this study are listed in the Key resources table (Table 1).

**Table 1:** Key resource table.

Reagent type (species) or resource	Designation	Source or reference	Identifiers	Additional information
Antibody	Anti-Actin, α-Smooth Muscle antibody, Mouse monoclonal	Sigma-Aldrich	A5228	WB (1:1000)
Antibody	Monoclonal Anti-β-Actin antibody produced in mouse, clone AC-15, from ascites fluid	Sigma-Aldrich	A5441	WB (1:5000)
Antibody	Anti-PARP-1 (46D11) Rabbit mAb	Cell Signaling Technology	9532S	WB (1:1000)
Antibody	Anti-mtTFA antibody (EPR12285)	Abcam	ab176558	WB (1:1000)
Antibody	Anti-COX2 (D-5)	Santa Cruz Biotechnology	sc-514489	WB (1:500)
Antibody	Anti-beta Actin antibody	Abcam	ab8226	WB (1:1000)
Antibody	Anti-Mouse IgG (H + L), AP Conjugate	Promega	S372B	WB (1:5000)
Antibody	Goat Anti-Mouse IgG H&L (HRP)	Abcam	ab6789	WB (1:4000)
Antibody	Goat Anti-Rabbit IgG H&L (HRP)	Abcam	ab205718	WB (1:4000)
Cell line	Normal fibroblasts (NFs)	Biobank and Beer’s laboratory, University Hospital Zurich		See Table S1
Cell line	Fibroblasts from basal cell carcinoma (BCC)	Biobank, University Hospital Zurich		See Table S1
Cell line	Fibroblasts from squamous cell carcinoma (SCC)	Beer’s laboratory, University Hospital Zurich		See Table S1
Commercial assay/kit	RNeasy Mini Kit	Qiagen	74104	
Commercial assay/kit	RNase-Free DNase	Qiagen	79254	
Commercial assay/kit	QIAprep Spin Miniprep Kit	Qiagen	27106	
Commercial assay/kit	Agencourt AMPure XP beads	Beckman Coulter	A63880	
Commercial assay/kit	High-Capacity cDNA Reverse Transcription Kit	ThermoFisher Scientific	4368814	
Commercial assay/kit	HOT FIREPol EvaGreen qPCR Mix	Solis Biodyne	08-25-00001	
Commercial assay/kit	GoTaq 2 × qPCR Master Mix	Promega	A6102	
Commercial assay/kit	RIPA Lysis and Extraction Buffer	ThermoFisher Scientific	89900	
Commercial assay/kit	Complete Mini EDTA-free Protease Inhibitor Cocktail	Roche	11836170001	
Commercial assay/kit	Pierce BCA protein assay kit	ThermoFisher Scientific	23225	
Commercial assay/kit	BCIP/NBT Color Development Substrate	Promega	S3771	
Commercial assay/kit	ECL Western Blotting Substrate	Promega	W1001	
Commercial assay/kit	Citrate Synthase Activity Assay Kit	Abcam	ab239712	
Commercial assay/kit	Seahorse XF Mito Stress Test Kit	Agilent	103015-100	
Commercial assay/kit	Mito Tracker Deep Red FM	ThermoFisher Scientific	M22426	
Software, algorithm	ImageJ	https://imagej.nih.gov/		
Software, algorithm	Fiji	https://imagej.net/software/fiji		
Software, algorithm	BioTek Gen5 (version 3.11)	Agilent		
Software, algorithm	Cellranger (version 3.1.0)	10× Genomics		
Software, algorithm	GraphPad Prism 9.0	https://www.graphpad.com		

### Citrate synthase (CS) assay

1.5 × 10^4^ NFs, BCC and SCC CAFs were seeded in 96 well-plates and assayed when a confluency of ~ 70–80% was reached. The activity of the citrate synthase enzyme, a proxy for cellular mitochondria content, was measured using the Citrate Synthase Activity Assay Kit (Abcam, UK) following manufacturer’s instructions. The colorimetric readings were acquired using a microplate Tecan instrument (Tecan, Infinite 200 PRO model, Switzerland) and normalized to a cell-free blank and to the amounts of harvested cells for each sample. Each condition was assayed in three biological replicates, and each sample was tested in three technical replicates.

### Measurement of reactive oxygen species (ROS)

1.5 × 10^4^ NFs, BCC and SCC CAFs were seeded in 96 well-plates and assayed at a confluency of ~ 70–80%. Culture medium was removed and replaced with 200 µl per well of a 50 µM 2′,7′-Dichlorofluorescin diacetate (DCF, Sigma Aldrich, USA) solution in PBS 1 ×. Plates were incubated for 30 min in the incubator to allow internalization of the chemical probe. The solution was removed, and 30 µl trypsin solution were added. Following 3–5 min incubation where detachment of the cells occurred, 120 µl PBS 1 × were added and fluorescence was acquired using a CytoFLEX flow cytometer (Beckman Coulter, USA). Data were normalized internally by gating. Cell-free blank controls and unstained controls treated in parallel but where DCF was omitted were as well performed and used as control for normalization. Each condition was assayed in three biological replicates, and each sample was tested in three technical replicates.

### Seahorse assays

Cellular oxygen consumption rate (OCR) and extra-cellular acidification rate (ECAR) were measured using the Seahorse Cell Metabolism Analyzer XF96. Cells from each patient-derived line were plated in 8 replicate wells at a density of 6000 cells/well in a 96-well Seahorse plate. After 24 h, media was changed to unbuffered XF assay media with 11 mmol/l glucose, 2 mmol/l glutamine and pyruvate at pH 7.4. Following basal measurement, oligomycin, FCCP, and rotenone/antimycin A were sequentially injected to achieve final concentrations of 1, 1.5, and 2 μM. Three blocks of 2 min mixing and 5 min measuring were used at basal and following each injection. After measurements, all plates were normalized to cell count using Hoechst staining and image-based nuclei counting.

### Imaging

Cells from each patient-derived line were plated in three replicate wells at a density of 3000 cells/well in a 96 well plate. After 24 h, cells were stained with 200 nM Mito Tracker Deep Red FM (ThermoFisher Scientific) and 200 ng/ml Hoechst in phenol-free DMEM with no serum for 20 min. Staining media was then replaced with phenol-free DMEM with 10% FBS and cells were imaged with a BioTek Cytation 5 using a 10 × objective. Mitochondrial area was segmented and quantified, and average Mito Tracker Red intensity was quantified within mitochondrial area using BioTek Gen5 (version 3.11).

### Analysis of single-cell sequencing data

Single cell RNA seq data on patient-matched normal and squamous cell carcinoma samples were obtained from GSE144240^27^. BAM files were downloaded from the NIH Sequence Resource Archive and converted to fastq files using the bamtofastq function in cellranger (version 3.1.0). Fastq files were then analyzed using the count function from cellranger. The GRCh38-2020-a reference from cellranger was used. Seurat^44^ objects were created using the outputs from cellranger. Normal and cancer cell data from the same patients were combined into a single Seurat object (Seurat version 2.3.4) in R (version 4.1.2). Cells with fewer than 200 RNA features and greater than 25% of reads assigned to mitochondrial DNA were filtered. Clusters were assigned a fibroblast identity based on expression of *THY1* and *COL1A1*. Differential expression between normal and cancer cells was determined using the FindMarkers function from Seurat.

### Statistical analyses

All values are expressed as mean ± standard deviation (SD). Information on number of biological replicates is reported in the figure legends. Differences in mean values between groups were analyzed using GraphPad Prism 9.0 (GraphPad Software, USA), and the adopted statistical analyses are indicated in the figure legends. All statistical tests were performed as two-tailed tests. All boxplots are styled as Tukey boxplots with the box representing median and first and third quartiles and whiskers representing the furthest point no more than 1.5*IQR from the box. In case of significant differences, the obtained *P* values are reported in the panels with *P* < 0.05 considered statistically significant (*P < 0.05. **P ≤ 0.01, ***P ≤ 0.001, ****P ≤ 0.0001). For statistical analyses including multiple genes or multiple clusters, multiple comparisons were controlled for using the Benjamini, Krieger and Yekutieli false detection rate correction. In these analyses uncorrected *P* values are reported and those passing false detection rate correction are indicated (*q < 0.05).

### Ethics

Isolation of cells from biopsies was approved by local ethics committee KEK-ZH-Nr. 2015-0198 and KEK-ZH-Nr. 2017-00688.

### Supplementary Information

Supplementary Information.

## Data Availability

All data generated during this study are available from the corresponding author upon reasonable request. The uncropped western blots used to generate the panels displayed in the main figures are included in the Supplementary Information file (Figs. S1–S3). The raw data relative to the single cell sequencing analyses depicted in Fig. 4 were originally from Ji et al., Cell 2020^27^ and available at Gene Expression Omnibus (GEO, https://www.ncbi.nlm.nih.gov/geo/) with the accession number GSE144240.

## References

[1] Sahai E, Astsaturov I, Cukierman E, DeNardo DG, Egeblad M, Evans RM, Fearon D, Greten FR, Hingorani SR, Hunter T (2020). A framework for advancing our understanding of cancer-associated fibroblasts. Nat. Rev. Cancer.

[2] Wang Z, Yang Q, Tan Y, Tang Y, Ye J, Yuan B, Yu W (2021). Cancer-associated fibroblasts suppress cancer development: The other side of the coin. Front. Cell Dev. Biol..

[3] Gunaydin G (2021). CAFs interacting with TAMs in tumor microenvironment to enhance tumorigenesis and immune evasion. Front. Oncol..

[4] Xiang H, Ramil CP, Hai J, Zhang C, Wang H, Watkins AA, Afshar R, Georgiev P, Sze MA, Song XS (2020). Cancer-associated fibroblasts promote immunosuppression by inducing ROS-generating monocytic MDSCs in lung squamous cell carcinoma. Cancer Immunol. Res..

[5] Simon T, Salhia B (2022). Cancer-associated fibroblast subpopulations with diverse and dynamic roles in the tumor microenvironment. Mol. Cancer Res..

[6] Lazard D, Sastre X, Frid MG, Glukhova MA, Thiery JP, Koteliansky VE (1993). Expression of smooth muscle-specific proteins in myoepithelium and stromal myofibroblasts of normal and malignant human breast tissue. Proc. Natl. Acad. Sci. USA.

[7] Rockey DC, Weymouth N, Shi Z (2013). Smooth muscle α actin (acta 2) and myofibroblast function during hepatic wound healing. PLoS One.

[8] Özdemir BC, Pentcheva-Hoang T, Carstens JL, Zheng X, Wu CC, Simpson TR, Laklai H, Sugimoto H, Kahlert C, Novitskiy SV (2014). Depletion of carcinoma-associated fibroblasts and fibrosis induces immunosuppression and accelerates pancreas cancer with reduced survival. Cancer Cell.

[9] Tan W, Zhang W, Strasner A, Grivennikov S, Cheng JQ, Hoffman RM, Karin M (2011). Tumour-infiltrating regulatory T cells stimulate mammary cancer metastasis through RANKL-RANK signalling. Nature.

[10] Comito G, Iscaro A, Bacci M, Morandi A, Ippolito L, Parri M, Montagnani I, Raspollini MR, Serni S, Simeoni L (2019). Lactate modulates CD4(+) T-cell polarization and induces an immunosuppressive environment, which sustains prostate carcinoma progression via TLR8/miR21 axis. Oncogene.

[11] Feig C, Jones JO, Kraman M, Wells RJ, Deonarine A, Chan DS, Connell CM, Roberts EW, Zhao Q, Caballero OL (2013). Targeting CXCL12 from FAP-expressing carcinoma-associated fibroblasts synergizes with anti-PD-L1 immunotherapy in pancreatic cancer. Proc. Natl. Acad. Sci. USA.

[12] Calon A, Lonardo E, Berenguer-Llergo A, Espinet E, Hernando-Momblona X, Iglesias M, Sevillano M, Palomo-Ponce S, Tauriello DVF, Byrom D (2015). Stromal gene expression defines poor-prognosis subtypes in colorectal cancer. Nat. Genet..

[13] Franco-Barraza J, Francescone R, Luong T, Shah N, Madhani R, Cukierman G, Dulaimi E, Devarajan K, Egleston BL, Nicolas E (2017). Matrix-regulated integrin α(v)β(5) maintains α(5)β(1)-dependent desmoplastic traits prognostic of neoplastic recurrence. eLife.

[14] Fontana GA, Gahlon HL (2020). Mechanisms of replication and repair in mitochondrial DNA deletion formation. Nucleic Acids Res..

[15] Nie H, Shu H, Vartak R, Milstein AC, Mo Y, Hu X, Fang H, Shen L, Ding Z, Lu J, Bai Y (2013). Mitochondrial common deletion, a potential biomarker for cancer occurrence, is selected against in cancer background: A meta-analysis of 38 studies. PLoS One.

[16] Corral-Debrinski M, Horton T, Lott MT, Shoffner JM, Flint Beal M, Wallace DC (1992). Mitochondrial DNA deletions in human brain: Regional variability and increase with advanced age. Nat. Genet..

[17] Quan C, Cho MK, Perry D, Quan T (2015). Age-associated reduction of cell spreading induces mitochondrial DNA common deletion by oxidative stress in human skin dermal fibroblasts: Implication for human skin connective tissue aging. J. Biomed. Sci..

[18] Zhu Q, Chen C, Yao J (2022). Kearns–Sayre syndrome with a novel large-scale deletion: A case report. BMC Ophthalmol..

[19] Wong LJC (2001). Recognition of mitochondrial DNA deletion syndrome with non-neuromuscular multisystemic manifestation. Genet. Med..

[20] Nie H, Chen G, He J, Zhang F, Li M, Wang Q, Zhou H, Lyu J, Bai Y (2016). Mitochondrial common deletion is elevated in blood of breast cancer patients mediated by oxidative stress. Mitochondrion.

[21] Chen T, He J, Shen L, Fang H, Nie H, Jin T, Wei X, Xin Y, Jiang Y, Li H (2011). The mitochondrial DNA 4,977-bp deletion and its implication in copy number alteration in colorectal cancer. BMC Med. Genet..

[22] Phillips AF, Millet AR, Tigano M, Dubois SM, Crimmins H, Babin L, Charpentier M, Piganeau M, Brunet E, Sfeir A (2017). Single-molecule analysis of mtDNA replication uncovers the basis of the common deletion. Mol. Cell.

[23] Berneburg M, Grether-Beck S, Kürten V, Ruzicka T, Briviba K, Sies H, Krutmann J (1999). Singlet oxygen mediates the UVA-induced generation of the photoaging-associated mitochondrial common deletion*. J. Biol. Chem..

[24] Gustafsson CM, Falkenberg M, Larsson N-G (2016). Maintenance and expression of mammalian mitochondrial DNA. Annu. Rev. Biochem..

[25] Herrmann GK, Russell WK, Garg NJ, Yin YW (2021). Poly(ADP-ribose) polymerase 1 regulates mitochondrial DNA repair in an NAD-dependent manner. J. Biol. Chem..

[26] D'Souza AR, Minczuk M (2018). Mitochondrial transcription and translation: Overview. Essays Biochem..

[27] Ji AL, Rubin AJ, Thrane K, Jiang S, Reynolds DL, Meyers RM, Guo MG, George BM, Mollbrink A, Bergenstråhle J (2020). Multimodal analysis of composition and spatial architecture in human squamous cell carcinoma. Cell.

[28] Shoffner JM, Lott MT, Voljavec AS, Soueidan SA, Costigan DA, Wallace DC (1989). Spontaneous Kearns–Sayre/chronic external ophthalmoplegia plus syndrome associated with a mitochondrial DNA deletion: A slip-replication model and metabolic therapy. Proc. Natl. Acad. Sci. USA.

[29] Moraes CT, DiMauro S, Zeviani M, Lombes A, Shanske S, Miranda AF, Nakase H, Bonilla E, Werneck LC, Servidei S (1989). Mitochondrial DNA deletions in progressive external ophthalmoplegia and Kearns–Sayre syndrome. N. Engl. J. Med..

[30] Porteous WK, James AM, Sheard PW, Porteous CM, Packer MA, Hyslop SJ, Melton JV, Pang CY, Wei YH, Murphy MP (1998). Bioenergetic consequences of accumulating the common 4977-bp mitochondrial DNA deletion. Eur. J. Biochem..

[31] Sturm G, Karan KR, Monzel AS, Santhanam B, Taivassalo T, Bris C, Ware SA, Cross M, Towheed A, Higgins-Chen A (2023). OxPhos defects cause hypermetabolism and reduce lifespan in cells and in patients with mitochondrial diseases. Commun. Biol..

[32] Nissanka N, Minczuk M, Moraes CT (2019). Mechanisms of mitochondrial DNA deletion formation. Trends Genet..

[33] Papadopoulos O, Champsas G (2020). Non-Melanoma Skin Cancer and Cutaneous Melanoma.

[34] Boukamp P (2005). Non-melanoma skin cancer: What drives tumor development and progression?. Carcinogenesis.

[35] Chen Y, Cairns R, Papandreou I, Koong A, Denko NC (2009). Oxygen consumption can regulate the growth of tumors, a new perspective on the Warburg effect. PLoS One.

[36] Semenza GL (2007). Hypoxia and cancer. Cancer Metastasis Rev..

[37] Jeon S, Jeon M, Choi S, Yoo S, Park S, Lee M, Kim I (2023). Hypoxia in skin cancer: Molecular basis and clinical implications. Int. J. Mol. Sci..

[38] Strittmatter GE, Garstkiewicz M, Sand J, Grossi S, Beer HD (2016). Human primary keratinocytes as a tool for the analysis of caspase-1-dependent unconventional protein secretion. Methods Mol. Biol..

[39] Grossi S, Fenini G, Hennig P, Di Filippo M, Beer HD (2020). Generation of knockout human primary keratinocytes by CRISPR/Cas9. Methods Mol. Biol..

[40] Purdie KJ, Pourreyron C, South AP (2011). Isolation and culture of squamous cell carcinoma lines. Methods Mol. Biol..

[41] Wahlsten A, Rutsche D, Nanni M, Giampietro C, Biedermann T, Reichmann E, Mazza E (2021). Mechanical stimulation induces rapid fibroblast proliferation and accelerates the early maturation of human skin substitutes. Biomaterials.

[42] Quispe-Tintaya W, White RR, Popov VN, Vijg J, Maslov AY (2013). Fast mitochondrial DNA isolation from mammalian cells for next-generation sequencing. Biotechniques.

[43] Schindelin J, Arganda-Carreras I, Frise E, Kaynig V, Longair M, Pietzsch T, Preibisch S, Rueden C, Saalfeld S, Schmid B (2012). Fiji: An open-source platform for biological-image analysis. Nat. Methods.

[44] Butler A, Hoffman P, Smibert P, Papalexi E, Satija R (2018). Integrating single-cell transcriptomic data across different conditions, technologies, and species. Nat. Biotechnol..

