# Supplementary material for: The mitochondrial DNA common deletion as a potential biomarker of cancer-associated fibroblasts from skin basal and squamous cell carcinomas
Source: Sci Rep. 2024 Jan 4;14:553. doi: 10.1038/s41598-023-50213-1 (PMC10766618; doi:10.1038/s41598-023-50213-1)
Supplement: Supplementary file 1 — Supplementary Information. [file 41598_2023_50213_MOESM1_ESM.docx]

**Supplementary information for:**

**The mitochondrial DNA common deletion as a potential biomarker of cancer-associated fibroblasts from skin basal and squamous cell carcinomas**

Gabriele A. Fontana^1^, Michael R. MacArthur^1^, Nadezhda Rotankova^1^, Michela Di Filippo^2,3^, Hans-Dietmar Beer^2,3^ and Hailey L. Gahlon^1*^

1. Department of Health Sciences and Technology, ETH Zurich, 8092 Zurich, Switzerland

2. Department of Dermatology, University Hospital Zurich, 8952 Schlieren, Switzerland

3. Faculty of Medicine, University of Zurich, 8032 Zurich, Switzerland

*Corresponding author: hailey.gahlon@hest.ethz.ch

**Table S1: Information on CAF origins**

| **BCC CAFs and NFs from healthy patients** | | | | |
| --- | --- | --- | --- | --- |
| **Donor #** | **Gender** | **Birth** | **Location** | **Histological diagnosis** |
| 171212F | F | 1939 | Unknown | No information, BCC patient |
| 180326F | M | 1928 | Ear | Basal cell carcinoma with nodular and basosquamous parts, extending into the deep dermis |
| 180427F | M | 1956 | Lumbar paravertebral | Basal cell carcinoma of the micronodular type, extending into the deep dermis |
| 180601F | Unknown | Unknown | Unknown | No information, BCC patient |
| 190626F | M | 1963 | Nose | Basal cell carcinoma of the nodular type. No evidence of perineural sheath infiltration. |
| 191203F | F | 1958 | Nose | Basal cell carcinoma with nodular and sclerosing parts |
| 210226F | M | 1941 | Mental recht | Basal cell carcinoma of micronodular type on actinically damaged skin |
| F170515F | F | 1972 | Abdomen | Healthy patient (control) |
| NHDF | Unknown | Unknown | Unknown | Healthy patient (control) |
|  | | | | |
| **SCC CAFs and patient-matched NFs** | | | | |
| **Donor #** | **Gender** | **Birth** | **Location** | **Histological diagnosis** |
| 11 | M | 1941 | Frontoparietal right | Moderately differentiated carcinoma spinocellulare with acantholysis, with infiltration of extracutaneous structures, extending into the subcutis, tumor thickness about 9mm |
| 19/11 | M | 1923 | Occipital | Ulcerated moderately differentiated carcinoma spinocellulare, reaching into the subcutis, tumor thickness 6mm |
| 30 | F | 1925 | Head left part | Excised shave with focal erosion and mixed-cell inflammation in the corium, without reliable evidence of a malignant invasive epithelial skin tumor |
| 31 | M | 1935 | Pectoral | Moderately differentiated carcinoma spinocellulare with infiltrative growth pattern, reaching to the cutis-subcutis border, tumor thickness 5mm |
| 32 | M | 1923 | Parietal rechts | Carcinoma spinocellulare in situ of the hyperplastic Bowen's disease type |
| 33 | M | 1928 | Ear helix right | Invasive, moderately differentiated spinocellular carcinoma, tumor thickness 5.6mm |
| 34 | M | 1935 | Rear part of the crown | Post-excision specimen with remnant of the previously diagnosed squamous cell carcinoma |
| 35 | M | 1949 | Forehead | Collision dermatosis from an irritated seborrheic keratosis and a hyperplastic actinic keratosis. no invasive spinocellular carcinoma detectable |
| 37 | M | 1936 | Head | Ulcerated moderately differentiated carcinoma spinocellulare of the Bowen carcinoma type, extending to the galea, tumor thickness 2.8mm |
| 38 | M | 1934 | Cheek right | Well to moderately differentiated carcinoma spinocellulare, partly infiltrative growing, reaching deep into the corium, tumor thickness 2mm |
| 39 | M | 1950 | Cheek right | Moderately differentiated carcinoma spinocellulare with acantholysis and with infiltration of extracutaneous structures, reaching down to the subcutis, Tumor 5mm thick |
| 40 | M | 1931 | Chest | Subsequent excision with remnants of the previously diagnosed, undifferentiated, infiltratively growing squamous cell carcinoma reaching to the subcutis, tumor thickness 4mm |
| 41 | M | 1928 | Forehead right | Invasive, moderately differentiated spinocellular carcinoma, tumor thickness 6mm, in collision with a nodular basal cell carcinoma, reaching into the middle corium |
| 42 | M | 1939 | Centro-frontoparietal right | Ulcerated, poorly differentiated carcinoma spinocellulare, reaching at least the middle corium, tumor thickness at least 2 mm |
| 46 | M | 1944 | Pretibial left | In situ SCC |

**Table S2: Primers used in this study**

| **Primers for qPCR-based analysis of gene level expression on cDNA** | | |
| --- | --- | --- |
| **Human gene and directionality** | **Sequence (5´ - 3´)** | **Source** |
| *ACTA2 FW* | GTGACGAAGCACAGAGCAAA | This study |
| *ACTA2 REV* | gggtgggatgctcttcag | This study |
| *ACTB FW* | TCCTTCCTGGGCATGGAGT | This study |
| *ACTB REV* | AGCACTGTGTTGGCGTACAG | This study |
| *APEX1 FW* | gaggagcatgatcaggaagg | This study |
| *APEX1 REV* | gctgttaccagcacaaacga | This study |
| *BRCA1 FW* | agagtccagctgctgctcat | This study |
| *BRCA1 REV* | ccctgctcacactttcttcc | This study |
| *CCN1 FW* | ACACCAAGGGGCTGGAAT | This study |
| *CCN1 REV* | gaaactttccccgttttgg | This study |
| *CXCL1 FW* | GGGAATTCACCCCAAGAAC | This study |
| *CXCL1 REV* | taactatgggggatgcagga | This study |
| *CXCL2 FW* | GCAGGGAATTCACCTCAAGA | This study |
| *CXCL2 REV* | cttaaccatgggcgatgc | This study |
| *DNA2 FW* | agctcttggcatgagtgaaag | This study |
| *DNA2 REV* | gcacggttaactgtacaacagc | This study |
| *ERCC2 FW* | ttctctgggctcgacgac | This study |
| *ERCC2 REV* | agtcgtacgggaagtagaccag | This study |
| *ERCC6 FW* | GGAGCAGAGGTGAAAATTGAA | This study |
| *ERCC6 REV* | ctcctggacaggcatgag | This study |
| *ERCC8 FW* | caagtcacagacaagaaatattagcag | This study |
| *ERCC8 REV* | agcacttgctgttgccaag | This study |
| *EXOG FW* | GGCTCCAGCAGGAAATAAC | This study |
| *EXOG REV* | caaaatcctgaggcacaatg | This study |
| *FAP FW* | GGAGATACTCTTACACAGCAACA | This study |
| *FAP REV* | tgaattggacgaggaagctca | This study |
| *FEN1 FW* | tcgaacttgctatgtaatttgtgtc | This study |
| *FEN1 REV* | aactcagctgattgccaggt | This study |
| *GAPDH FW* | ACGGATTTGGTCGTATTGGG | This study |
| *GAPDH REV* | TGATTTTGGAGGGATCTCG | This study |
| *HUMANIN FW* | cgagggttcagctgtctctt | This study |
| *HUMANIN REV* | ggcaggtcaatttcactggt | This study |
| *IL1B FW* | GGCAATGAGGATGACTTGT | This study |
| *IL1B REV* | ggagattcgtagctggatgc | This study |
| *IL6 FW* | GGCTGAAAAAGATGGATGC | This study |
| *IL6 REV* | gctctggcttgttcctcac | This study |
| *LIG3 FW* | caacacgaagacccagatca | This study |
| *LIG3 REV* | ggtacacatcaccgtggaaa | This study |
| *MGME1 FW* | gactgaaaagccccaaagtct | This study |
| *MGME1 REV* | gcgtgtcctcctttcaggta | This study |
| *MPG FW* | tttacggcatgtacttctgcat | This study |
| *MPG REV* | atggtctccagaccttccag | This study |
| *MRE11 FW* | acaacctggaagctcagtgg | This study |
| *MRE11 REV* | ttaatacgcagcaaaccaaca | This study |
| *mtRNA UTR FW* | ctttgattcctgcctcatcc | This study |
| *mtRNA UTR FW* | tgatgtctgtgtggaaagtgg | This study |
| *MT-ATP6 FW* | ccacaatcctaggcctaccc | This study |
| *MT-ATP6 REV* | gggatcaatagagggggaaa | This study |
| *MT-ATP8 FW* | gcctactcattcaaccaatagc | This study |
| *MT-ATP8 REV* | tcagtagaattagaattgtgaagatga | This study |
| *MT-CO1 FW* | atcctaccaggcttcggaat | This study |
| *MT-CO1 REV* | cggaggtgaaatatgctcgt | This study |
| *MT-CO2 FW* | ccatccctacgcatccttta | This study |
| *MT-CO2 REV* | ggtcgcctggttctaggaat | This study |
| *MT-CO3 FW* | cccgctaaatcccctagaag | This study |
| *MT-CO3 REV* | atggtgaagggagactcgaa | This study |
| *MT-CYB FW* | tatccgccatcccatacatt | This study |
| *MT-CYB REV* | ggtgattcctagggggttgt | This study |
| *MT-ND1 FW* | tgaagtcaccctagccatca | This study |
| *MT-ND1 REV* | ggttcggttggtctctgcta | This study |
| *MT-ND2 FW* | aagcaaccgcatccataatc | This study |
| *MT-ND2 REV* | tcagaagtgaaagggggcta | This study |
| *MT-ND3 FW* | ccacaactcaacggctacat | This study |
| *MT-ND3 REV* | ttgtagggctcatggtaggg | This study |
| *MT-ND4 FW* | ctcgctaacctcgccttacc | This study |
| *MT-ND4 REV* | agtgagccccattgtgttgt | This study |
| *MT-ND4L FW* | tcgctcacacctcatatcct | This study |
| *MT-ND4L REV* | gccatatgtgttggagattga | This study |
| *MT-ND5 FW* | cgcttccccacccttacta | This study |
| *MT-ND5 REV* | gcgagggctgtgagttttag | This study |
| *MT-ND6 FW* | tctgaattttgggggaggtt | This study |
| *MT-ND6 REV* | ccacagcaccaatcctacct | This study |
| *MT-RNR1 FW* | AAACGCTTAGCCTAGCCACA | This study |
| *MT-RNR1 REV* | CTTTACGCCGGCTTCTATTG | This study |
| *MT-RNR2 FW* | actttgcaaggagagccaaa | This study |
| *MT-RNR2 REV* | tggacaaccagctatcacca | This study |
| *MUTYH FW* | GTGTGTATCAGGGCCAACAG | This study |
| *MUTYH REV* | gactgcacggagaggacac | This study |
| *OGG1 FW* | TGTCACCTACCATGGCTTCC | This study |
| *OGG1 REV* | aggcccagcttcctgaga | This study |
| *NEIL1 FW* | agctgcgcctgatactgag | This study |
| *NEIL1 REV* | gctgaaaagagccggacat | This study |
| *NEIL2 FW* | gggcagcagtaagaagctaca | This study |
| *NEIL2 REV* | tgcaggaccaacctcacc | This study |
| *NTHL1 FW* | aggtgctgctgtcactgatg | This study |
| *NTHL1 REV* | tctgcaggatgctgtcca | This study |
| *PARP1 FW* | GGGATGACCAGCAGAAAGTC | This study |
| *PARP1 REV* | ctgccttgctaccaattcc | This study |
| *PDGFRA FW* | ATCCGGCGTTCCTGGTCTTA | This study |
| *PDGFRA REV* | actgccagctcacttcactc | This study |
| *PNKP FW* | gacagcatctttgtgggagac | This study |
| *PNKP REV* | aaggttgagggcaaacagg | This study |
| *POLG FW* | CAGGTACCACCCTGGAGTC | This study |
| *POLG REV* | ccctgttcgagacagtgctt | This study |
| *POLG2 FW* | CACGAACTTTTACACATGTATCC | This study |
| *POLG2 REV* | cagagagaacacaaggaacc | This study |
| *POLRMT FW* | CATGTACAACGCCGTGATG | This study |
| *POLRMT REV* | ggcatccttcaccatgaataa | This study |
| *PTGS2 FW* | CAGCACTTCACGCATCAGTT | This study |
| *PTGS2 REV* | cgctgtctagccagagtttc | This study |
| *RAD23A FW* | atgcggcaggtgattcag | This study |
| *RAD23A REV* | agggccttcaactgtaaaagc | This study |
| *RAD51 FW* | gggaattagtgaagccaaagc | This study |
| *RAD51 REV* | tggtgaaacccattggaact | This study |
| *RNASEH1 FW* | GGCCTTTGTCAGGAAATCTG | This study |
| *RNASEH1 REV* | ctttcgcctccgattcttgt | This study |
| *S100A1 FW* | CTGAGCTGGAGACGGCGATG | This study |
| *S100A1 REV* | ctccttcatcaccttgtccacagc | This study |
| *SPP1 FW* | GCCGAGGTGATAGTGTGGTT | This study |
| *SPP1 REV* | ccatgtgtgaggtgatgtcc | This study |
| *SSBP1 FW* | CATGAGTCCGAAACAACTACCA | This study |
| *SSBP1 REV* | acagggtcctgacccactc | This study |
| *TEFM FW* | GAGAAAGCTCCTCAAACCAG | This study |
| *TEFM REV* | caattcttcgagtaccaaaaacg | This study |
| *TFAM FW* | tgcaacttctgtggaagcat | This study |
| *TFAM REV* | gaatcaggaagttccctcca | This study |
| *TFB2M FW* | aaatttggacgaatagaagtaaatatg | This study |
| *TFB2M REV* | AGTCTGGATTTCCGGGATCT | This study |
| *TOP1MT FW* | CACAACAAAGGAGGTTTTCC | This study |
| *TOP1MT REV* | ccaggctcttgatgacttcc | This study |
| *TOP3A FW* | GCCCAAGAGCAAGTGGCG | This study |
| *TOP3A REV* | cctcatggtttctttagcattt | This study |
| *TWNK FW* | GGACCTGCCCCTCTATTTC | This study |
| *TWNK REV* | catagacgtagactgcatgttgc | This study |
| *UNG FW* | gctgagtgccgagcagtt | This study |
| *UNG REV* | tgcttcttccagctctctcc | This study |
| *VIM FW* | TTTCCAAGCCTGACCTCACG | This study |
| *VIM REV* | tcagtggactcctgctttgc | This study |
| *XRCC4 FW* | gaggtaggatccggaagtgg | This study |
| *XRCC4 REV* | gaaacaaggtggattctgctta | This study |
| *XRCC6 FW* | gccttgtcctcagccagtta | This study |
| *XRCC6 REV* | cctcgacttatgtcgggtaga | This study |
| *YBX1 FW* | CAATGTAAGGAACGGATATGG | This study |
| *YBX1 REV* | ggtgtacaaatacatcttccttgg | This study |
|  | | |
| **Primers for qPCR-based analysis of mtDNA species** | | |
| **Target and directionality** | **Sequence (5´ - 3´)** | **Source** |
| *mtDNA total FW* | TAGCCCTAAACCTCAACAGT | [22] |
| *mtDNA total REV* | TGCGCTTACTTTGTAGCCTTCAT | [22] |
| *mtDNA CD FW* | TTCCTCATCACCCAACTAAAAA | [22] |
| *mtDNA CD REV* | TTCGATGATGTGGTCTTTGG | [22] |
| *mtDNA undeleted FW* | tcgatgatgtggtctttgga | This study |
| *mtDNA undeleted FW* | catctgtacccacgccttct | This study |
| *ACTB genomic FW* | TCACCCACACTGTGCCCATCTACGA | [22] |
| *ACTB genomic REV* | CAGCGGAACCGCTCATTGCCAATGG | [22] |

**Table S3: Fold Change qPCR Data from Figures 2A and 2B**

qPCR data: BCC CAFs over NFs controls

|  | **Fold change over NF controls** | | | | **Fold change over NF controls** | | |
| --- | --- | --- | --- | --- | --- | --- | --- |
| **Gene** | **BCC CAF1** | **BCC CAF2** | **BCC CAF3** | | **average** | **st dev** |  |
| *LIG3* | 0.796916 | 0.424107 | 0.775125 |  | 0.665383 | 0.209235 |  |
| *MGME1* | 1.915207 | 1.220523 | 5.019348 |  | 2.718359 | 2.02276 |  |
| *TFAM* | 1.033472 | 1.862835 | 9.629761 |  | 4.175356 | 4.74182 |  |
| *PARP1* | 7.260153 | 1.105731 | 12.08378 |  | 6.816555 | 5.502452 |  |
| *TWNK* | 0.823591 | 1.93858 | 2.928171 |  | 1.896781 | 1.052913 |  |
| *POLG* | 2.571304 | 1.523617 | 4.148242 |  | 2.747721 | 1.321176 |  |
| *POLG2* | 1.23328 | 0.761808 | 8.83053 |  | 3.608539 | 4.528516 |  |
| *RNASEH1* | 0.848214 | 0.507859 | 6.419674 |  | 2.591916 | 3.319301 |  |
| *SSBP1* | 0.624165 | 0.392292 | 1.370783 |  | 0.795747 | 0.511313 |  |
| *POLRMT* | 1.140764 | 0.632878 | 1.918528 |  | 1.230723 | 0.647529 |  |
| *TEFM* | 1.45902 | 0.662044 | 4.642816 |  | 2.254627 | 2.106271 |  |
| *TFB2M* | 2.989698 | 1.310393 | 6.233317 |  | 3.511136 | 2.502542 |  |
| *TOP1MT* | 0.775125 | 0.415379 | 4.034808 |  | 1.74177 | 1.993958 |  |
| *TOP3A* | 0.794159 | 0.387562 | 1.263565 |  | 0.815095 | 0.438377 |  |
| *OGG1* | 0.876606 | 0.534033 | 3.999999 |  | 1.803546 | 1.909881 |  |
| *MUTYH* | 1.726081 | 0.799683 | 25.6787 |  | 9.401486 | 14.10408 |  |
| *UNG* | 4.806544 | 5.063026 | 14.723 |  | 8.197524 | 5.652685 |  |
| *MPG* | 2.571304 | 3.738605 | 23.95907 |  | 10.08966 | 12.02543 |  |
| *NEIL1* | 0.218772 | 0.183965 | 7.821796 |  | 2.741511 | 4.399691 |  |
| *NEIL2* | 1.265757 | 0.126306 | 15.83451 |  | 5.74219 | 8.758752 |  |
| *NTHL1* | 0.226487 | 0.121371 | 2.755855 |  | 1.034571 | 1.491602 |  |
| *APEX1* | 0.843816 | 0.468136 | 0.716978 |  | 0.67631 | 0.191113 |  |
| *ERCC6* | 538.3871 | 589.1536 | 1025.776 |  | 717.7722 | 267.9441 |  |
| *ERCC8* | 110.2779 | 66.25696 | 396.1766 |  | 190.9038 | 179.1289 |  |
| *RAD23A* | 32.89964 | 125.3658 | 121.5158 |  | 93.26041 | 52.30939 |  |
| *ERCC2* | 2.505329 | 1.574616 | 37.66102 |  | 13.91365 | 20.57108 |  |
| *FEN1* | 0.205898 | 0.125 | 4.69134 |  | 1.674079 | 2.613337 |  |
| *EXOG* | 0.917004 | 0.466516 | 1.515717 |  | 0.966412 | 0.526342 |  |
| *PNKP* | 0.630689 | 0.349896 | 2.353813 |  | 1.111466 | 1.085026 |  |
| *YBX1* | 0.697372 | 0.336808 | 0.773782 |  | 0.602654 | 0.233378 |  |
| *MRE11* | 1.079228 | 0.547147 | 2.211461 |  | 1.279279 | 0.85 |  |
| *BRCA1* | 0.189465 | 0.089622 | 1.693491 |  | 0.657526 | 0.89856 |  |
| *DNA2* | 0.698581 | 0.402623 | 4.899043 |  | 2.000082 | 2.514931 |  |
| *XRCC6* | 0.52395 | 0.542427 | 0.866037 |  | 0.644138 | 0.192392 |  |
| *XRCC4* | 3.300078 | 0.141365 | 9.366435 |  | 4.269293 | 4.688284 |  |
| *RAD51* | 0.246131 | 0.113637 | 5.071808 |  | 1.810525 | 2.82513 |  |

qPCR data: SCC CAFs over NFs controls

|  | **Fold change over NF controls** | | | | **Fold change over NF controls** | |
| --- | --- | --- | --- | --- | --- | --- |
| **Gene** | **SCC CAF1** | **SCC CAF2** | **SCC CAF3** | | **average** | **st dev** |
| *LIG3* | 7.46 | 8.19 | 9.435 |  | 8.361667 | 0.998628 |
| *MGME1* | 12.275 | 8.965 | 10.58 |  | 10.60667 | 1.655161 |
| *TFAM* | 17.96 | 15.72 | 16.215 |  | 16.63167 | 1.176694 |
| *PARP1* | 8.485 | 7.11 | 9.04 |  | 8.211667 | 0.993609 |
| *TWNK* | 8.44 | 9.775 | 10.47333 |  | 9.562778 | 1.033146 |
| *POLG* | 9.705 | 11.68667 | 11.89 |  | 11.09389 | 1.207102 |
| *POLG2* | 9.445 | 10.795 | 12.23 |  | 10.82333 | 1.392716 |
| *RNASEH1* | 6.245 | 7.98 | 9.68 |  | 7.968333 | 1.71753 |
| *SSBP1* | 6.245 | 7.205 | 8.856667 |  | 7.435556 | 1.32101 |
| *POLRMT* | 7.05 | 8.43 | 9.56 |  | 8.346667 | 1.257073 |
| *TEFM* | 8.695 | 9.76 | 11.22333 |  | 9.892778 | 1.269386 |
| *TFB2M* | 8.42 | 9.59 | 11.215 |  | 9.741667 | 1.403659 |
| *TOP1MT* | 7.633333 | 9.785 | 10.215 |  | 9.211111 | 1.383207 |
| *TOP3A* | 7.565 | 8.52 | 9.76 |  | 8.615 | 1.100579 |
| *OGG1* | 9.015 | 10.585 | 11.075 |  | 10.225 | 1.076151 |
| *MUTYH* | 8.705 | 9.915 | 9.755 |  | 9.458333 | 0.657292 |
| *UNG* | 8.43 | 10.76 | 11.54 |  | 10.24333 | 1.618096 |
| *MPG* | 15.55 | 18.595 | 24.74 |  | 19.62833 | 4.681331 |
| *NEIL1* | 10.14 | 11.73667 | 17.3 |  | 13.05889 | 3.75867 |
| *NEIL2* | 22.105 | 25.205 | 16.28 |  | 21.19667 | 4.531303 |
| *NTHL1* | 10.345 | 12.84 | 13.42 |  | 12.20167 | 1.633863 |
| *APEX1* | 5.81 | 7.545 | 8.975 |  | 7.443333 | 1.584947 |
| *ERCC6* | 5.64 | 7.985 | 8.275 |  | 7.3 | 1.444896 |
| *ERCC8* | 9.133333 | 10.41 | 11.335 |  | 10.29278 | 1.105504 |
| *RAD23A* | 17.535 | 13.715 | 22.635 |  | 17.96167 | 4.47528 |
| *ERCC2* | 10.89 | 18.405 | 14.34 |  | 14.545 | 3.761692 |
| *FEN1* | 6.955 | 9.68 | 10.31 |  | 8.981667 | 1.783188 |
| *EXOG* | 6.62 | 9.29 | 9.525 |  | 8.478333 | 1.613648 |
| *PNKP* | 6.94 | 9.6 | 9.675 |  | 8.738333 | 1.557854 |
| *YBX1* | 2.423333 | 4.15 | 5.855 |  | 4.142778 | 1.715845 |
| *MRE11* | 7.855 | 9.73 | 10.915 |  | 9.5 | 1.542911 |
| *BRCA1* | 8.04 | 10.695 | 12 |  | 10.245 | 2.017988 |
| *DNA2* | 9.58 | 10.86 | 13.00333 |  | 11.14778 | 1.729715 |
| *XRCC6* | 8.33 | 8.52 | 10.02 |  | 8.956667 | 0.925761 |
| *XRCC4* | 13.115 | 14.62667 | 15.72 |  | 14.48722 | 1.308086 |
| *RAD51* | 9.65 | 12.03 | 13.5 |  | 11.72667 | 1.942842 |

**
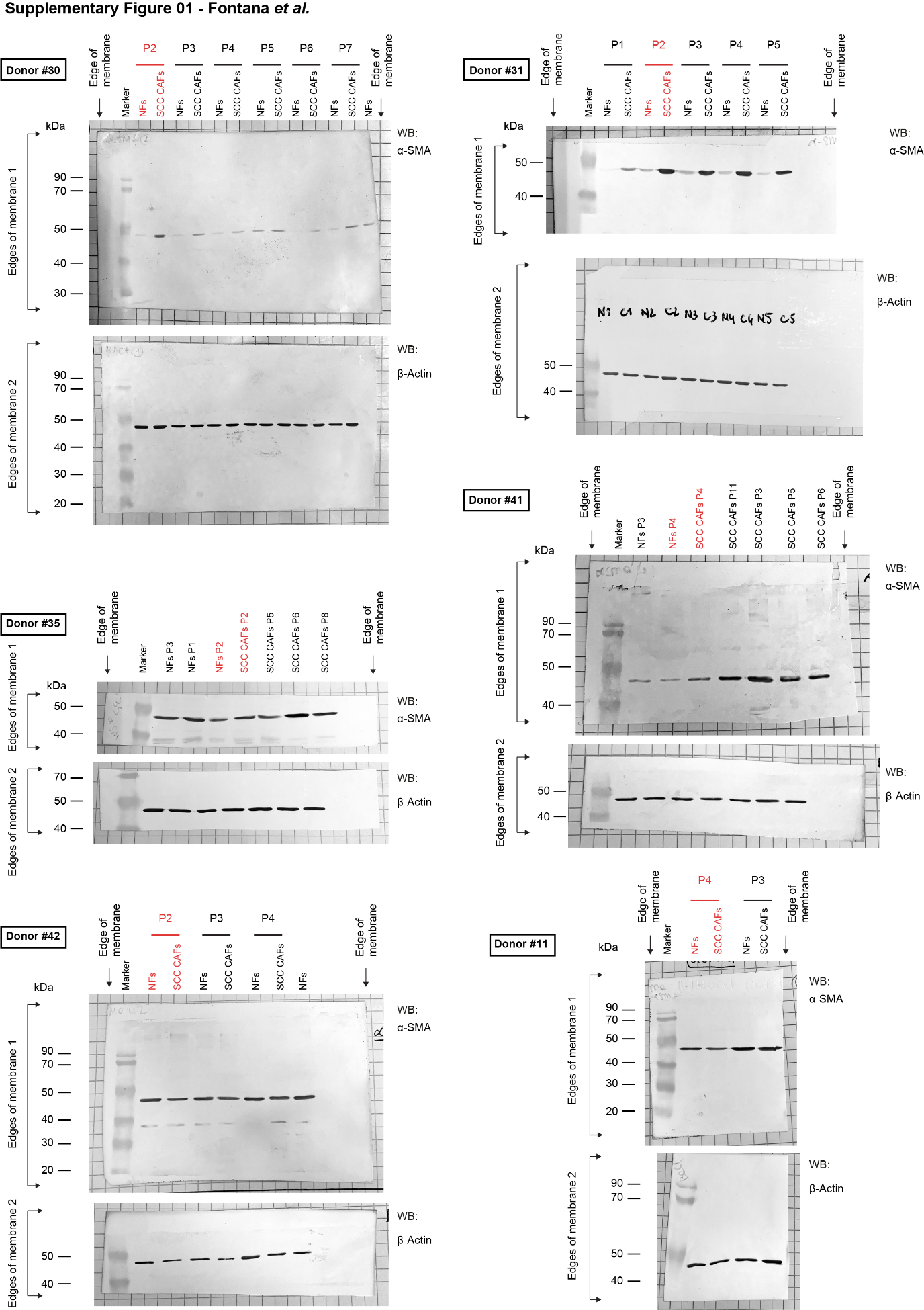
**

**Supplementary Figure 1: Uncropped western blots.**

Uncropped western blots used to assemble the panels in Figure 1B. The lanes corresponding to the samples shown in Figure 1B are labelled in red, while lanes marked in black are relative to samples not shown. The culture passage (P) when NFs and SCC CAFs samples were processed is indicated for each lane. Donor number is indicated on the top left-hand side of each blot pair and refers to Table S1. The edges of western blot membranes are indicated by arrows on the top (left and right edges) and on the left side (top and bottom edges) of the panels. As the predicted molecular weights of human α-SMA and β-Actin are similar and secondary antibodies used for their detection in western blots would cross-react, the same samples were run on two independent gels and processed in parallel. Membranes were cut in either in the ~90/~20 or ~70/~30 kDa regions and hybridized with the antibodies indicated on the right side. Molecular weights are depicted on the left side.

**
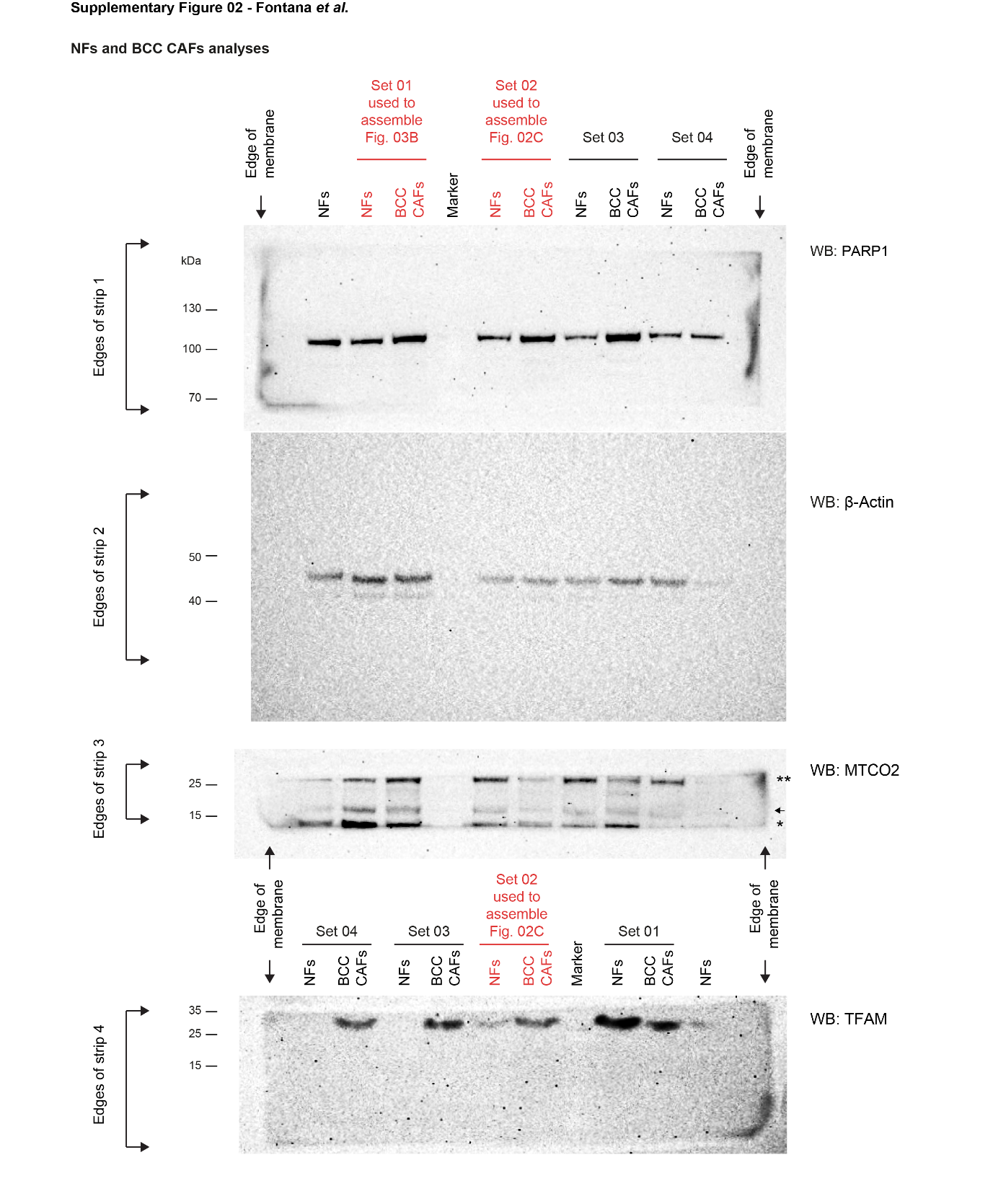
**

**Supplementary Figure 2: Uncropped western blots.**

Uncropped western blots used to assemble the panels in Figure 2C and 3B and relative to NFs and BCC CAFs analyses. The lanes corresponding to the samples shown in Figure 2C and 3B are labelled in red, while lanes marked in black are relative to samples not shown. Four biological NFs-BCC CAFs sets (Set 01-04, indicated on the top part of the membranes) were analyzed. The edges of western blot membranes are indicated by arrows on the top/bottom (left and right edges) and on the left side (top and bottom edges) of the panels. The predicted molecular weights of human TFAM and MTCO2 are similar, and the MT-CO2 antibody displayed unspecific bands in western blots; thus, to allow the concomitant detection of both proteins, the same samples were run on two independent gels (Strip 3 for MTCO2 detection and Strip 4 for TFAM detection, note different loading order) and processed in parallel. Membranes were cut in the ~130/~70 kDa (Strip 1), ~60/~35 kDa (Strip 2) and ~35/~15 kDa (Strip 3 and 4) regions and hybridized with antibodies against PARP1, β-Actin, MTCO2 or TFAM antibodies, respectively. Molecular weights are indicated on the left sides and the antibodies used for hybridization are listed on the right. On the right-hand side of strip 3 an arrowhead indicates the MTCO2 band, while a single black asterisk (shown in Figure 3B) and double asterisks (not shown in Figure 3B) designate cross-reactive unspecific bands. PARP1, TFAM and β-Actin blots are shown in Figure 2C, while MTCO2 and β-Actin blots are shown in Figure 3B.

**
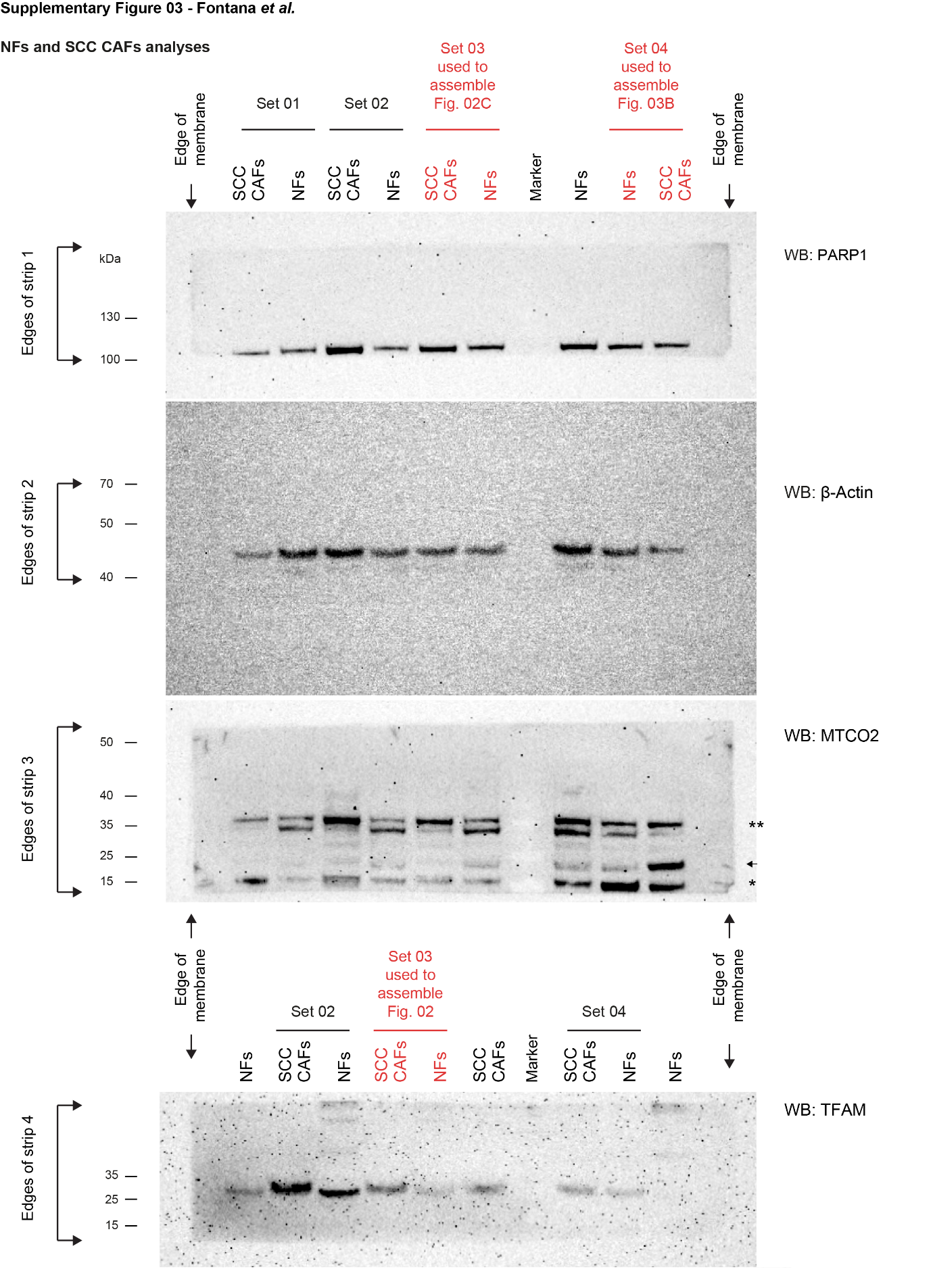
**

**Supplementary Figure 3: Uncropped western blots.**

Uncropped western blots used to assemble the panels in Figure 2C and 3B and relative to NFs and SCC CAFs analyses. The lanes corresponding to the samples shown in Figure 2C and 3B are labelled in red, while lanes marked in black are relative to samples not shown. Four biological NFs-SCC CAFs sets (Set 01-04, indicated on the top part of the membranes) were analyzed. The edges of western blot membranes are indicated by arrows on the top/bottom (left and right edges) and on the left side (top and bottom edges) of the panels. The predicted molecular weights of human TFAM and MTCO2 are similar, and the MT-CO2 antibody displayed unspecific bands in western blots; thus, to allow the concomitant detection of both proteins, the same samples were run on two independent gels (Strip 3 for MTCO2 detection and Strip 4 for TFAM detection) and processed in parallel. Membranes were cut in the ~130/~90 kDa (Strip 1), ~70/~40 kDa (Strip 2) and ~50/~15 /~35/~15 kDa (Strip 3 and 4) regions and hybridized with antibodies against PARP1, β-Actin, MTCO2 or TFAM antibodies, respectively. Due to scarcity of biological Set 01 samples its analyses were limited to PARP1, TFAM and β-Actin expression (Strip 1, 2 and 3 respectively); samples relative to biological Set 02-03-04 were further analyzed for TFAM expression (Strip 4, note different loading order). Molecular weights are indicated on the left side and the antibodies used for hybridization are listed on the right. On the right-hand side of strip 3 an arrowhead indicates the MTCO2 band, while a single asterisk (shown in Figure 3B) and double asterisks (not shown in Figure 3B) designate cross-reactive unspecific bands. PARP1, TFAM and β-Actin blots are shown in Figure 2C, while MTCO2 and β-Actin blots are shown in Figure 3B.
